# Irisin exhibits neuroprotection by preventing mitochondrial damage in Parkinson’s disease

**DOI:** 10.1038/s41531-023-00453-9

**Published:** 2023-01-31

**Authors:** Xi Zhang, Sutong Xu, Yong Hu, Qiulu Liu, Chenming Liu, Huazhen Chai, Yuping Luo, Lingjing Jin, Siguang Li

**Affiliations:** 1 Key Laboratory of Spine and Spinal Cord Injury Repair and Regeneration of Ministry of Education, Orthopedic Department of Tongji Hospital, Tongji University School of Medicine, Shanghai, China; 2 Department of rehabilitation Medicine, Huashan Hospital, Fudan University, Shanghai, China; 3 Department of Neurology and Neurological Rehabilitation, Shanghai Yangzhi Rehabilitation Hospital, Tongji University School of Medicine, Shanghai, China; 4 Department of Neurology, Tongji Hospital, Tongji University School of Medicine, Shanghai, China

**Keywords:** Parkinson's disease, Parkinson's disease, Cellular neuroscience

## Abstract

Exercise has been proposed as an effective non-pharmacological management for Parkinson’s disease (PD) patients. Irisin, a recently identified myokine, is increased by exercise and plays pivotal roles in energy metabolism. However, it remains unknown whether irisin has any protective effects on PD. Here, we found that serum irisin levels of PD patients were markedly elevated after 12-week regular exercise, which had a positive correlation with improved balance function scored by Berg Balance Scale. Treatment with exogenous irisin could improve motor function, and reduce dopaminergic neurodegeneration in PD models. Meanwhile, irisin could reduce cell apoptosis by renovating mitochondrial function in PD models, which was reflected in decreased oxidative stress, increased mitochondrial complex I activity and mitochondrial content, increased mitochondrial biogenesis, and repaired mitochondrial morphology. Furthermore, irisin regulated the aforementioned aspects by upregulating downstream Akt signaling pathway and ERK1/2 signaling pathway through integrin receptors rather than directly targeting mitochondria. With the use of small-molecule inhibitors, it was found that irisin can reduce apoptosis, restore normal mitochondrial biogenesis, and improve mitochondrial morphology and dynamic balance in PD models by activating Akt signaling pathway and ERK1/2 signaling pathway. And irisin reduced oxidative stress via activating ERK1/2 signaling pathway. The results revealed that exogenous irisin conferred neuroprotection relieving apoptosis and oxidative stress, restraining mitochondrial fragmentation, and promoting mitochondrial respiration and biogenesis in PD models, and irisin exerted the aforementioned effects by activating Akt signaling pathway and ERK1/2 signaling pathway. Thus, peripherally delivered irisin might be a promising candidate for therapeutic targeting of PD.

## Introduction

Parkinson’s disease (PD) is an age-related and the second most common neurodegenerative disorder characterized by the loss of dopaminergic neurons in the substantia nigra (SN) and the proteinaceous aggregates in neurons called Lewy bodies (LBs)^1^. And according to the post-mortem examination of human SN tissues, functional loss of mitochondrial complex I could be observed in the SN of PD patients^2^. Although the pathogenesis of PD is regulated by both environmental and genetic factors, accumulating evidences has highlighted the key role of mitochondrial dysfunction that is involved in both sporadic and familial PD. Firstly, multiple environmental toxins increase PD risk by inhibiting mitochondrial complex I, like rotenone and 1-methyl-4-phenyl-1,2,3,6-tetrahydropyridine (MPTP)^3,4^. Then a number of PD-related genes synergistically lead to mitochondrial dysfunction. For example, mutation in PARK2, PARK6, PARK7, PARK8, PARK17, PARK22 is identified as autosomal recessive or dominant form of PD, all of which encode proteins that are involved in maintaining mitochondrial health^5–8^. Moreover, alpha-synuclein encoded by the first PD-linked gene SNCA, a component of Lewy bodies, has been shown to accumulate in mitochondria, interfering with complex I function and mediating mitochondrial dysfunction^5,9^. Besides, the latest evidence suggests that disruption of mitochondrial complex I induces progressive PD^10^. In a word, mitochondrial dysfunction lies at the heart of PD.

Fibronectin-domain III containing 5 (FNDC5) is a glycosylated type 1 membrane protein, and has been identified as an exercise-regulated factor^11^. And FNDC5 is cleaved and the N-terminal portion of it is released into the circulation; the secreted form has been named irisin. Irisin contains 112 amino acids, is heavily glycosylated and is 100% conserved between mouse and human^11,12^. Currently, irisin could be regarded as an extremely promising myokine since the effect of peripheral irisin injection in mice was very similar to that led by endurance exercise^13^, and exercise has been proposed as a non-pharmacological management for people who are in the early stages of PD or people who are experiencing balance or motor function problems^14,15^. The structure of irisin and its elevation with endurance exercise in humans has been confirmed using tandem mass spectrometry of human plasma^16^. In bones and fat tissues, irisin mediates its effects via αV integrin receptors and following FAK/Akt signaling pathway^17^. Since the discovery as a PGC-1α-dependent myokine in 2012, irisin has been given great expectations on the management of numerous diseases^18–22^. The roles of irisin in improving cognitive, memory and learning function in Alzheimer’s disease (AD) have already been described in previous studies, and elevation of circulating irisin levels by peripheral delivery could pass through the blood–brain barrier and result in enrichment of central irisin which ameliorated both the cognitive deficit and neuropathology in AD mouse models^23,24^.

Nonetheless, the function of irisin in PD remains largely unknown. In the present study, we detected the serum irisin level of PD patients before and at the end of 12-week exercise, and analyzed the association between peripheral irisin levels and motor symptoms. We show that regular rehabilitation exercise can increase the serum irisin of PD patients which is positively associated with the improvement of balance function. And we also indicate that exogenous irisin exhibits neuroprotection by preventing mitochondrial damage in PD models. Therefore, this study aimed to elucidate the effect of irisin in PD and its potential mechanisms.

## Results

### Regular rehabilitation exercise increases the serum irisin of PD patients which is positively associated with the improvement of balance function

To assess the relationship of irisin and exercise in PD patients, 23 patients were involved in this study. As shown in Table 1 and Supplementary Fig. 1a–c, we showed that 12-week regular exercise could improve motor function and balance of PD patients according to scores of UPDRSIII and BBS. After 12-week regular exercise, the serum irisin levels of PD patients (2.11 ± 0.53 μg/mL) were notably higher than those before (1.89 ± 0.62 μg/mL). In addition, a positive correlation was observed between increased serum irisin levels and improved BBS scores of PD patients (*R* = 0.49, *p* = 0.018, Supplementary Fig. 1e), whereas increased serum irisin levels had no association with improved UPDRSIII scores (*R* = 0.−0.087, *p* = 0.69, Supplementary Fig. 1d).

**Table 1:** Demographic data, motor function score, and laboratory results of patients with Parkinson’s disease.

	Group		
	Before exercise (*n* = 23)	After exercise (*n* = 23)	*p*-value
Age (years)	68.08 ± 7.07	68.08 ± 7.07	–
Gender			
Male	15 (65.2%)	15 (65.2%)	–
Female	8 (34.8%)	8 (34.8%)	–
Hoehn and Yahr Stage (median)	2.5 (1–3)	–	–
Levodopa dose (mg/day)	310.79 ± 206.89	–	–
Irisin (μg/mL)	1.89 ± 0.62	2.11 ± 0.53	0.041*
UPDRSIII Score	31.11 ± 11.07	25.70 ± 9.08	0.083
BBS	50.96 ± 2.96	53.13 ± 2.54	0.012*

UPDRSIII Parkinson’s Disease Rating Scale Motor Examination, BBS Berg Balance Scale.

### Peripherally delivered irisin improves motor function and dopaminergic neurodegeneration in MPTP treated mice

**Fig. 1:**
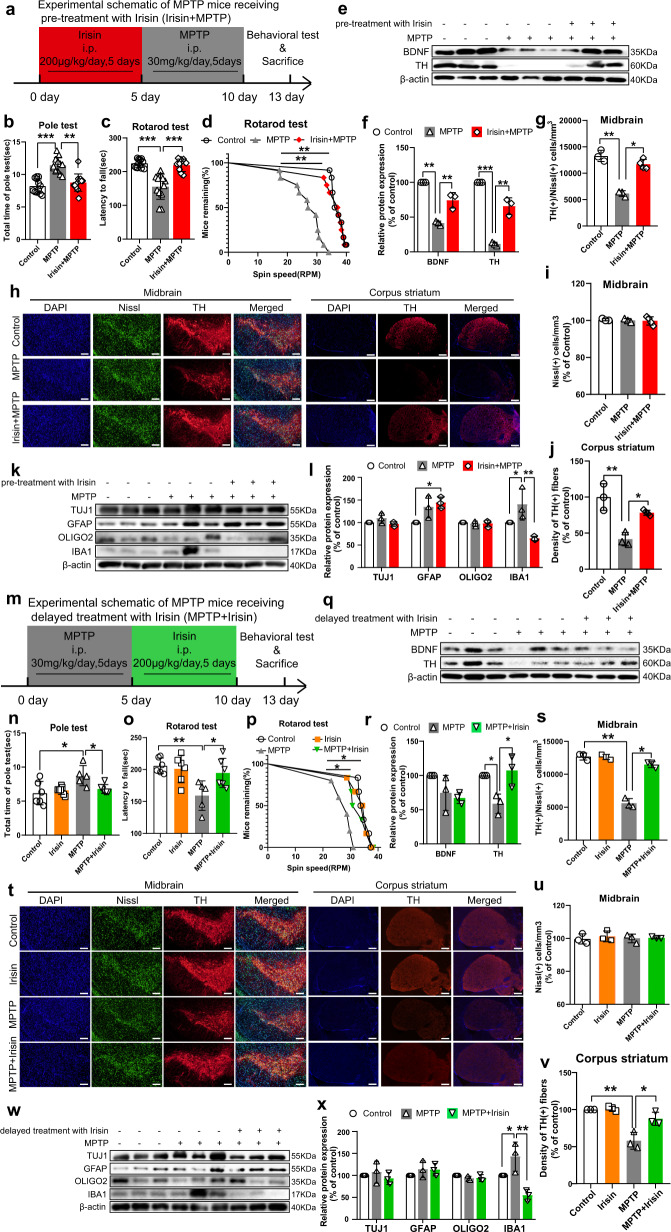
The impaired motor function and dopaminergic neurodegeneration in PD models induced by neurotoxins could be improved by peripheral irisin pretreatment or treatment. Treatment with irisin in mice was conducted by i.p. (200 μg/kg, 5 days) at the beginning or ending of MPTP treatment (30 mg/kg, 5 days). **a** Experimental schematic of MPTP treated mice receiving pre-treatment with irisin (intraperitoneal injection, i.p.) (WT *n* = 9, MPTP *n* = 11, irisin-MPTP *n* = 9). **b**–**d** The motor activity in the pole test (**b**) and rotarod test (**c**, **d**) for MPTP treated mice receiving pre-treatment with irisin. **e**, **f** Western blot showing BDNF and TH expression in the midbrain of MPTP treated mice receiving pre-treatment with irisin. **g**–**j** Confocal image and relative quantitative analysis of coronal sections showing TH expression in the striatum and TH (+) / Nissl (+) cells in the midbrain. Scale bar, 200 μm. **k**, **l** Western blot showing TUJ1, GFAP, OLIGO2 and IBA1 expression in the midbrain of MPTP treated mice receiving pre-treatment with irisin. **m** Experimental schematic of MPTP treated mice receiving delayed treatment with irisin (i.p.) (WT *n* = 6, WT-irisin *n* = 6, MPTP *n* = 6, MPTP-irisin *n* = 6). **n–p** The motor activity in the pole test (**n**) and rotarod test (**o**, **p**) for MPTP treated mice receiving delayed treatment with irisin. **q**, **r** Western blot showing BDNF and TH expression in the midbrain of MPTP treated mice receiving delayed treatment with irisin. **s**–**v** Confocal image and relative quantitative analysis of coronal sections showing TH expression in the striatum and TH (+) / Nissl (+) cells in the midbrain. Scale bar, 200 μm. **w**, **x** Western blot showing TUJ1, GFAP, OLIGO2 and IBA1 expression in the midbrain of MPTP treated mice receiving delayed treatment with irisin. β-actin is used as a loading control. All data are presented as mean ± SEM (*n* = 3). ^∗^*p* < 0.05, ^∗∗^*p* < 0.01, and ^∗∗∗^*p* < 0.001.

For further study, we selected the most commonly used MPTP treated mice to simulate the motor impairment and pathological injury of PD. To examine the role of irisin in PD, irisin was peripherally delivered before or after the establishment of MPTP treated mice to explore its effect on neuroprotection and treatment (Fig. 1a, m), in which irisin injection dose was the median dose reported in literature^13,17,20,22,23,25–29^. To evaluate the effect of irisin in MPTP-induced motor disfunction, we evaluated behavioral tests in mice. Compared with MPTP-treated mice, both the pre-treatment and delayed treatment of irisin reduced total time of pole test and increased remain time on the rotarod (Fig. 1b–d, n–p), which showed that irisin could improve MPTP-induced balance disorders. In addition to the behavior test, the pre-treatment and delayed treatment of irisin markedly alleviated the MPTP-induced damage of TH (+) fibers in corpus striatum, and that of TH (+) and Nissl (+) cells number, and signally restored protein levels of TH in midbrain (Fig. 1e–j, q–v). Both the pre-treatment and delayed treatment of irisin could significantly decreased protein levels of IBA1 which was induced by MPTP in midbrain (Fig. 1k, l, w, x). However, the changes of TUJ1, GFAP and OLIGO2 were not obvious (Fig. 1 k, l, w, x). Notably, impaired BDNF level in MPTP-treated mice could be restored by pre-treatment of irisin rather than delayed treatment of irisin (Fig. 1e, f, q, r). Besides, peripherally delivered irisin could not enhance motor function of WT mice, as well as TH (+) fibers or TH (+) and Nissl (+) cells in brains of WT mice (Fig. 1m–o).

### Irisin reduces apoptosis in PD models induced by neurotoxins

**Fig. 2:**
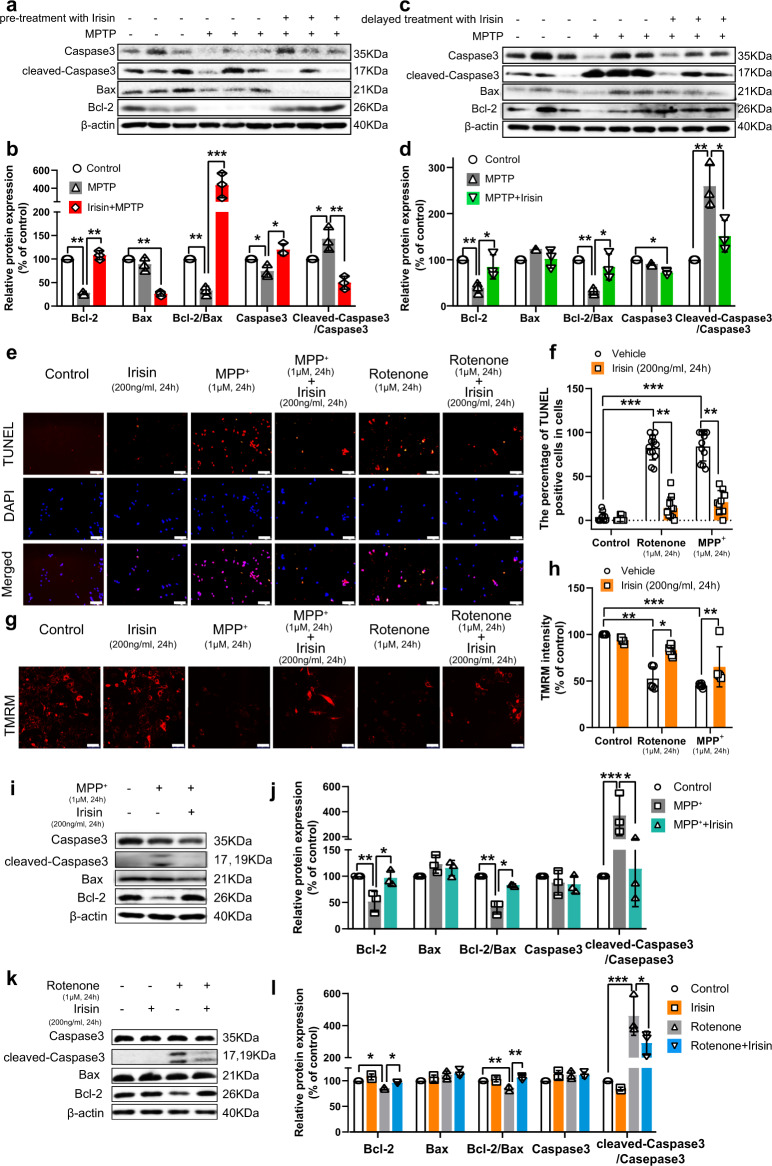
Peripheral irisin induces apoptosis in PD models induced by neurotoxins. SH-SY5Y cells were seeded and incubated until 80% cell density. And the cells were separately treated with vehicle, rotenone (1 μM, 24 h), irisin (200 ng/ml, 24 h), MPP^+^ (1 μM, 24 h), rotenone (1 μM, 24 h) and irisin (200 ng/ml, 24 h) co-treatment, and MPP^+^ (1 μM, 24 h) and irisin (200 ng/ml, 24 h) co-treatment. **a**, **b** Western blot showing the quantification of cleaved-caspase3/caspase3 ratio, Bax, Bcl-2, and Bcl-2/Bax ratio in the midbrain of MPTP treated mice receiving pre-treatment with irisin. **c**, **d** Western blot showing the quantification of cleaved-caspase3/caspase3 ratio, Bax, Bcl-2, and Bcl-2/Bax ratio in the midbrain of MPTP treated mice receiving delayed treatment with irisin. **e**, **f** Confocal image of cell coverslips showing TUNEL ( + ) cell number in various groups of in vitro experiments. Scale bar, 25 μm. **g**, **h** Confocal image of TMRM showing mitochondrial membrane potential (MMP) in various groups of in vitro experiments. Scale bar, 25 μm. **i**, **j** Western blot showing the quantification of cleaved-caspase3/caspase3 ratio, Bax, Bcl-2, and Bcl-2/Bax ratio in SH-SY5Y cells treated by MPP^+^ and irisin. **k**, **l** Western blot showing the quantification of cleaved-caspase3/caspase3 ratio, Bax, Bcl-2, and Bcl-2/Bax ratio in SH-SY5Y cells treated by rotenone and irisin. All data are presented as mean ± SEM (*n* ≥ 3). ^∗^*p* < 0.05, ^∗∗^*p* < 0.01, and ^∗∗∗^*p* < 0.001.

Mounting evidence indicated that different morphological types of cell death coexisted in the brain of PD patients, all of which could result from the activation of common mitochondrion-dependent apoptosis^30–33^. To evaluate the role of irisin on apoptosis, we supplemented in vitro experiments of irisin on MPP^+^-induced and rotenone-induced SH-SY5Y cell models, and we constructed time-dose curves to determine the optimal dose and treatment time of these reagents (Supplementary Fig. 2a–e). For the role of irisin on apoptosis, we demonstrated that irisin administration markedly upregulated Bcl-2 expression and Bcl-2/Bax ratio, and reduced the activation and cleavage of caspase3 in PD mice and cell models (Fig. 2a–d, i–l). And combined with TUNEL and MMP results (Fig. 2e–h), irisin management reduced the activation mitochondrion-dependent apoptosis and recovered cell viability (Supplementary Fig. 2d, e) in in vivo and in vitro experiments.

### Irisin reduces oxidative stress in PD models induced by neurotoxins

**Fig. 3:**
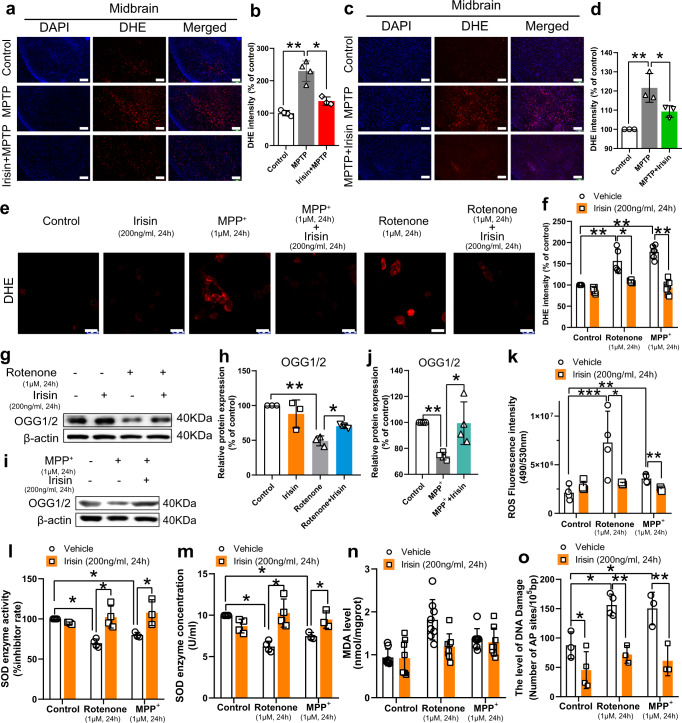
Irisin decreases oxidative stress in PD models induced by neurotoxins. **a**, **b** DHE fluorescence staining and its fluorescence intensity in midbrain of MPTP treated mice receiving pre-treatment with irisin. Scale bar, 200 μm. **c**, **d** DHE fluorescence staining and its fluorescence intensity in midbrain of MPTP treated mice receiving delayed treatment with irisin. Scale bar, 200 μm. **e**, **f** DHE fluorescence staining and its fluorescence intensity in various groups of in vitro experiments. Scale bar, 30 μm. **g**, **h** Western blot showing the quantification of OGG1/2 in SH-SY5Y cells treated with irisin and MPP^+^. **i**, **j** Western blot showing the quantification of OGG1/2 in SH-SY5Y cells treated with irisin and rotenone. **k** DCFH-DA assay showing ROS level in various groups of in vitro experiments. **l**, **m** SOD enzyme activity and SOD enzyme concentration in various groups of in vitro experiments. **n** MDA level in various groups of in vitro experiments. **o** The level of AP sites showing the level of DNA damage in various groups of in vitro experiments. All data are presented as mean ± SEM (*n* ≥ 3). ^∗^*p* < 0.05, ^∗∗^*p* < 0.01, and ^∗∗∗^*p* < 0.001.

Given that oxidative stress is related to apoptosis and mitochondrial dysfunction in PD^34^, we examined the effect of irisin on oxidative stress, with ROS and MDA levels indicating the oxidants level, SOD activity representing the antioxidants level, and AP site and repair enzyme, OGG1/2, reflecting the level of ROS-induced DNA injury^35,36^. According to DCFH-DA assay and DHE staining, irisin notably reduced ROS accumulation in PD mice and cell models (Fig. 3a–f, k), whereas MDA was not involved in MPTP or rotenone induced oxidative stress (Fig. 3n). Consistently, delivery of irisin also alleviated the ROS-induced DNA damage and its repair enzyme damage (Fig. 3g–j, o). And irisin also restored the impaired SOD activity (Fig. 3l, m). Taking the three aforementioned aspects into consideration, we illustrated that irisin significantly reduced oxidative stress and its injury in PD models.

### Irisin restores the impaired mitochondrial function in PD models induced by neurotoxins

**Fig. 4:**
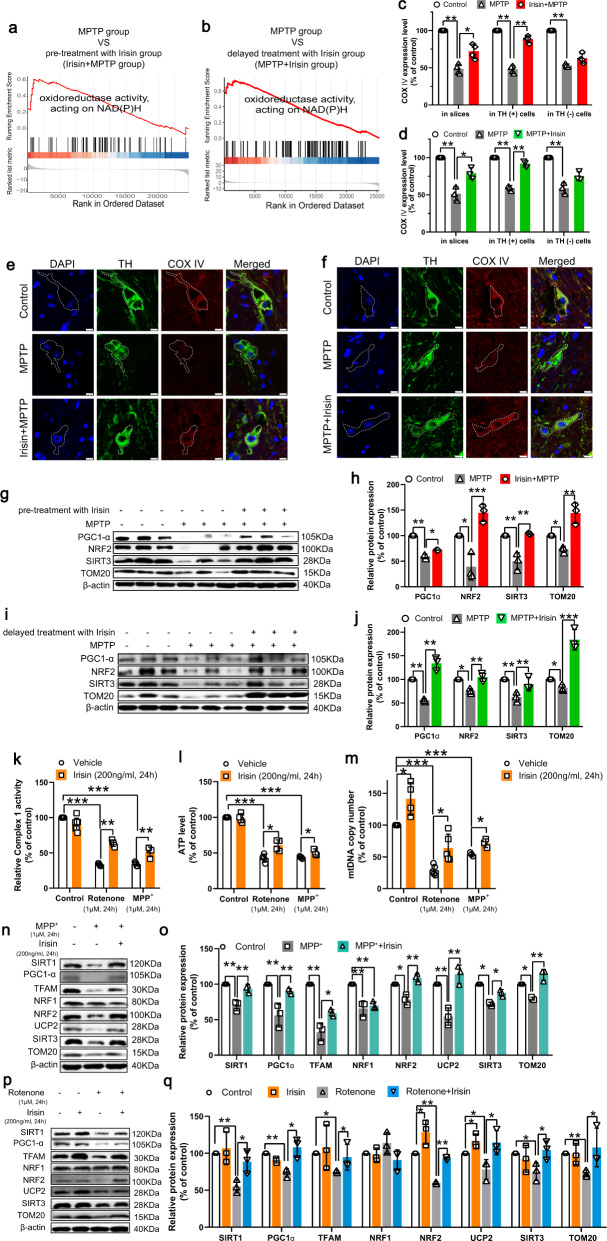
Irisin recovers impaired mitochondrial function in PD models induced by neurotoxins. **a** GSEA analysis of “oxidoreductase activity, acting on NAD(P)H” in midbrain in MPTP treated mice receiving pre-treatment with irisin. **b** GSEA analysis of “oxidoreductase activity, acting on NAD(P)H” in midbrain in MPTP treated mice receiving delayed treatment with irisin. **c**, **e** Confocal image and relative quantitative analysis showing COX IV and TH expression of dopaminergic neurons in MPTP treated mice receiving pre-treatment with irisin. Scale bar, 10 μm. **d**, **f** Confocal image and relative quantitative analysis showing COX IV and TH expression of dopaminergic neurons in MPTP treated mice receiving delayed treatment with irisin. Scale bar, 10 μm. **g**, **h** Western blot showing the quantification of PGC-1α, NRF-2, SIRT3 and TOM20 in each group of MPTP treated mice receiving pre-treatment with irisin. **i**, **j** Western blot showing the quantification of PGC-1α, NRF-2, SIRT3 and TOM20 in each group of MPTP treated mice receiving delayed treatment with irisin. **k** Mitochondrial complex I activity in various groups of in vitro experiments. **l** ATP level in various groups of in vitro experiments. **m** mtDNA copy number in various groups of in vitro experiments. **n**, **o** Western blot showing the quantification of SIRT1, PGC-1α, NRF1, NRF-2, UCP2, SIRT3, TFAM and TOM20 in each group of SH-SY5Y cells treated by irisin and MPP^+^. **p**, **q** Western blot showing the quantification of SIRT1, PGC-1α, NRF1, NRF-2, UCP2, SIRT3, TFAM and TOM20 in each group of SH-SY5Y cells treated by irisin and rotenone. All data are presented as mean ± SEM (*n* ≥ 3). ^∗^*p* < 0.05, ^∗∗^*p* < 0.01, and ^∗∗∗^*p* < 0.001. GSEA, gene set enrichment analysis.

To further investigate the role of irisin in PD, we performed transcriptomic analysis by RNA-seq. Gene set enrichment analysis (GSEA) of RNA-Seq data from MPTP treated mice with irisin pretreatment and treatment respectively identified plentiful pathways. Of note, several pathways associated with NADH activity and metabolism in MPTP treated mice with irisin pretreatment overlapped with those pathways identified in MPTP treated mice with irisin treatment (Supplementary Fig. 3a, c, Fig. 4a, b). It is well known that NAD(H) activity and regeneration can reflect mitochondrial complex I activity, which play a critical role in mitochondrial respiration and biogenesis, and ATP synthesis^37,38^. As a result, we demonstrated that MPTP- or rotenone-induced complex I inhibition and ATP synthesis damage were alleviated by irisin (Fig. 4k, l), which indicated that irisin could promote the restoration of mitochondrial function in PD models induced by neurotoxins. Besides, NAD^+^/NADH ratio could activate Sirtuin-1 (SIRT1) that is an NAD^+^-dependent enzyme deeply involved in mitochondrial biogenesis^39^. Thus, we evaluated expression levels of proteins associated with mitochondrial biogenesis, and mitochondria number assessed by translocase of outer mitochondrial membrane 20 (TOM20) and COX IV. In short, we observed that irisin recovered damaged mitochondria number in dopaminergic neurons (Fig. 4c–f) and elevated the inhibited expression levels of SIRT1, PGC-1α, nuclear respiratory factors 2 (NRF-2) and mitochondrial transcription factor A (TFAM), TOM20 in PD models except nuclear respiratory factors 1 (NRF-1) (Fig. 4g–j, n–q), and mtDNA copy number was renewed (Fig. 4m). Therefore, irisin effectually restored the impaired mitochondrial function in PD models induced by neurotoxins.

### Irisin regains thwarted mitochondrial dynamics and morphology in PD models induced by neurotoxins

**Fig. 5:**
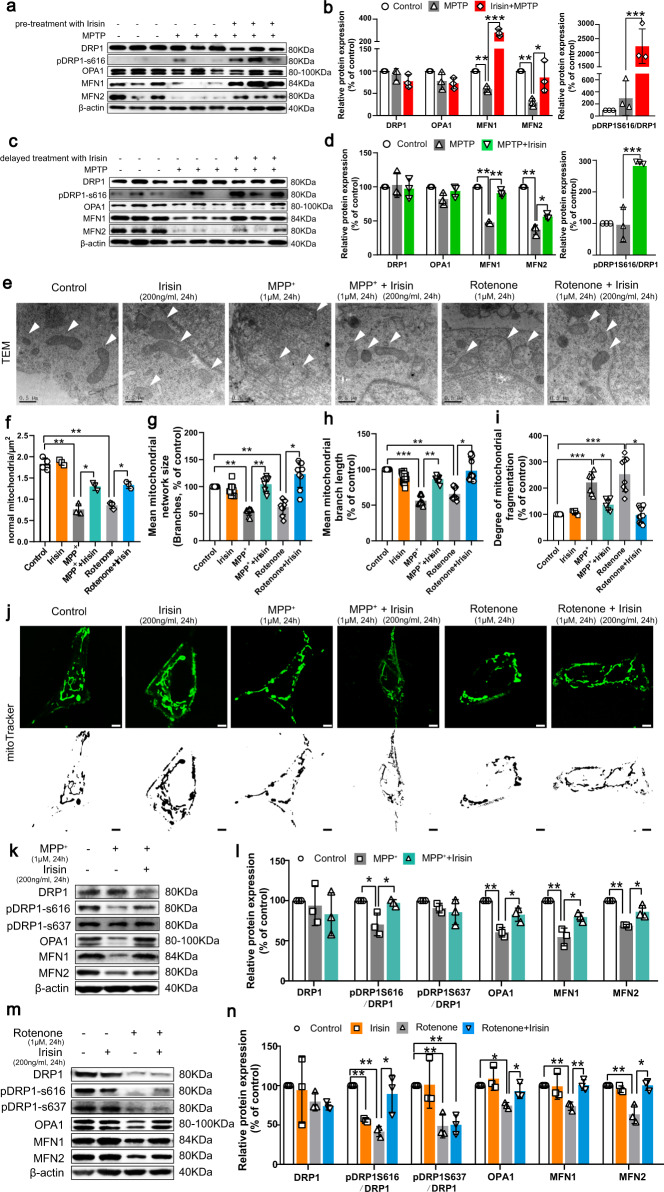
Irisin regains thwarted mitochondrial fusion and fission and morphology in PD models induced by neurotoxins. **a**, **b** Western blot showing the quantification of DRP1, pDRP1-S616/DRP1 ratio, OPA1, MFN1and MFN2 in each group of MPTP treated mice receiving pre-treatment with irisin. **c**, **d** Western blot showing the quantification of DRP1, pDRP1-S616/DRP1 ratio, OPA1, MFN1and MFN2 in each group of MPTP treated mice receiving delayed treatment with irisin. **e**, **f** Transmission electron microscopy image and relative quantitative analysis of SH-SY5H cells showing mitochondrial morphology in various groups of in vitro experiments (magnification 40,000×). Scale bar, 0.5 μm. **g**–**j** mitoTracker fluorescence staining and its quantification analysis of mitochondrial fragmentation in various groups of in vitro experiments. Scale bar, 2 μm. **k**, **l** Western blot showing the quantification of DRP1, pDRP1-S616/DRP1 ratio, pDRP1-S637/DRP1 ratio, OPA1, MFN1and MFN2 in each group of SH-SY5Y cells treated by irisin and MPP^+^. **m**, **n** Western blot showing the quantification of DRP1, pDRP1-S616/DRP1 ratio, pDRP1-S637/DRP1 ratio, OPA1, MFN1and MFN2 in each group of SH-SY5Y cells treated by irisin and rotenone. All data are presented as mean ± SEM (*n* ≥ 3). ^∗^*p* < 0.05, ^∗∗^*p* < 0.01, and ^∗∗∗^*p* < 0.001.

Mitochondrial function depends on mitochondrial morphology regulated by continuous fusion and fission cycles^40,41^. To explicitly evaluate mitochondrial structural features, mitoTracker and TEM were used to observe mitochondria. According to mitoTracker and TEM results, we ascertained that mitochondrion under normal conditions exhibited an interconnected tubular network structure, dense matrix and orderly packed cristae structure, whereas the structure in in vitro experiments established by MPP^+^ or rotenone appeared as mitochondrial fragmentation (short separate tubes or swollen tubes), and those mitochondria exhibited a swollen form, sparse matrix and disrupted cristae (Fig. 5e–j). These results suggested disorder of mitochondrial dynamics, including dysregulated fission-fusion processes. We used western blot to indicate that dynamin-related protein 1 (Drp-1) and its phosphorylation, optic atrophy protein 1 (OPA1), Mitofusin 1 and 2 (MFN1 and MFN2), mitochondrial fission- and fusion-related proteins, were significantly downregulated in the MPTP, MPP^+^ or rotenone-treated group compared with the vehicle group (Fig. 5a–d, k–n). However, pre-treatment and delayed treatment with exogenous irisin markedly restored the expression levels of these proteins in PD models induced by neurotoxins (Fig. 5a–d, k–n). Our data further confirmed that irisin could upregulate these proteins that mediate mitochondrial dynamics, and alleviate the damage of mitochondrial form.

### Irisin confers protection by binding to αV integrin receptors to activate signaling pathways rather than directly targeting mitochondria after transported into the cells

**Fig. 6:**
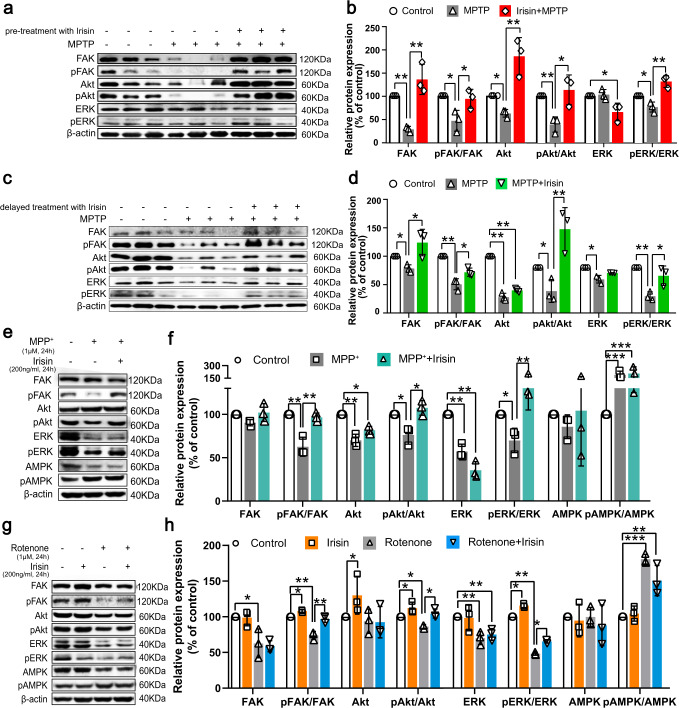
Irisin activate integrin receptor signaling pathway. **a**, **b** Western blot showing the quantification of the p-FAK/FAK ratio, p-Akt/Akt ratio, p-ERK/ERK ratio in each group of MPTP treated mice receiving pre-treatment with irisin. **c**, **d** Western blot showing the quantification of the p-FAK/FAK ratio, p-Akt/Akt ratio, p-ERK/ERK ratio in each group of MPTP treated mice receiving delayed treatment with irisin. **e**, **f** Western blot showing the quantification of the p-FAK/FAK ratio, p-Akt/Akt ratio, p-ERK/ERK ratio, and p-AMPK/AMPK ratio in each group of SH-SY5Y cells treated by irisin and MPP^+^. **g**, **h** Western blot showing the quantification of the p-FAK/FAK ratio, p-Akt/Akt ratio, p-ERK/ERK ratio, and p-AMPK/AMPK in each group of SH-SY5Y cells treated by irisin and rotenone. All data are presented as mean ± SEM (*n* ≥ 3). ^∗^*p* < 0.05, ^∗∗^*p* < 0.01, and ^∗∗∗^*p* < 0.001.

It was recently identified that αVβ5 integrins were receptors for irisin in bone and fat cells^17^ and its downstream “focal adhesion” pathway was enriched and significantly regulated in our RNA-seq analyses (Supplementary Fig. 3b, d)^42–44^. Furthermore, “PI3K-Akt signaling pathway” and “MAPK signaling pathway” were observably enriched in our RNA-seq analyses (Supplementary Fig. 3b, d), which could be activated by upstream focal adhesion kinase (FAK). To verify whether irisin could active aforementioned signaling pathways, we detected the activation of core molecules in PI3K-Akt signaling pathway and in ERK1/2 signaling pathway, since ERK1/2 signaling pathway acts as one of the most important part of MAPK pathway^45^. Compared with vehicle group, irisin could activate the phosphorylation of FAK, Akt and ERK1/2, indicating the increased activation levels of these pathways (Fig. 6g, h). Relatively, peripheral irisin restored the ratio of phospho-FAK (Tyr397) /FAK, phospho-Akt (Ser473) / Akt, and phospho-ERK1 (T202/Y204) + ERK2 (T185/Y187) /ERK1/2 (Fig. 6a–h) in MPTP-, MPP^+^- or rotenone-treated mice and SH-SY5Y cells. Interestingly, irisin did not change the expression and activation of AMP-activated protein kinase (AMPK), which regulated cell metabolism^26,46^. In a word, irisin could reactivate PI3K-Akt signaling pathway and MAPK signaling pathway in MPTP treated mice and toxins-treated SH-SY5Y cells.

**Fig. 7:**
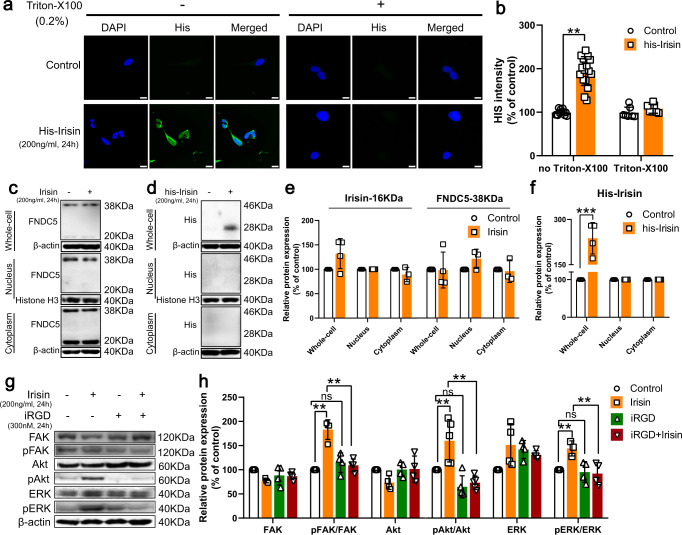
Irisin confers neuroprotection by binding to αV integrin receptors to activate signaling pathways rather than directly targeting mitochondria after transported into the cell. **a**, **b** Confocal image and relative quantitative analysis of cell coverslips showing his-flag expression in irisin-treated SH-SY5Y cell. Scale bar, 100 μm. **c**–**f** The SH-SY5Y cells were treated with irisin, his-irisin or control for 24 h. The cell lysates were analyzed by western blotting with the indicated antibodies. Data shows the quantification of irisin (**c**, **e**) or exogenous irisin (**d**, **f**) in whole-cell, cytoplasm and nucleus. **g**, **h** The SH-SY5Y cells were treated with irisin, iRGD peptides, or irisin and iRGD peptides for 24 h. The cell lysates were analyzed by western blotting with the indicated antibodies. Data shows the quantification of the p-FAK/FAK ratio, p-Akt / Akt ratio, p-ERK/ERK ratio. All data are presented as mean ± SEM (*n* ≥ 3). ns *p* > 0.05, ^∗^*p* < 0.05, ^∗∗^*p* < 0.01, and ^∗∗∗^*p* < 0.001.

Although αV integrins were identified as receptors for irisin in bone and fat cells, numerous evidences revealed that irisin worked by directly targeting mitochondria^27,28^. Since the activation of integrin receptor signaling pathway (PI3K-Akt signaling pathway and MAPK signaling pathway) and the restoration of mitochondrial function appeared simultaneously in our research, it was necessary to clarify the relationships between them. To ensure the binding site of Irisin, we performed IF staining in SH-SY5Y cells treated by his-irisin in the presence or absence of Triton-X100 (0.2%, 15 min) to penetrate cell membrane. Then we did not observe His staining in triton-X 100-treated cells, but faint His staining could be observed in cells without triton-X 100 treatment (Fig. 7a, b). These results supported that exogenous irisin could not be transported in to cells. To further verify this point, we respectively detected levels of irisin in whole-cell, cytoplasm and nucleus (Fig. 7c, e). Because currently commercial FNDC5 antibody could not distinguish irisin and FNDC5, his-irisin was utilized to identify exogenous irisin, and exogenous irisin was observed in his-irisin-treated whole-cell rather than in cytoplasm and nucleus (Fig. 7d, f). In short, exogenous irisin could not be transported into cells to target mitochondria but target membrane. To ensure whether irisin combined with integrins αV receptors to act, we used iRGD peptide to block integrin αV complexes^47^. As shown in Fig. 10g, h, iRGD peptide notably restrained irisin-induced phos-phorylation of FAK, Akt, and ERK1/2 (Fig. 7g, h). In brief, exogenous irisin confers the aforementioned neuroprotection by binding to αV integrin receptors to activate signaling pathways rather than directly targeting mitochondria after transported into the cell.

### Irisin prevents apoptosis and oxidative stress via activation of Akt and ERK1/2 signaling pathways in PD models induced by neurotoxins

**Fig. 8:**
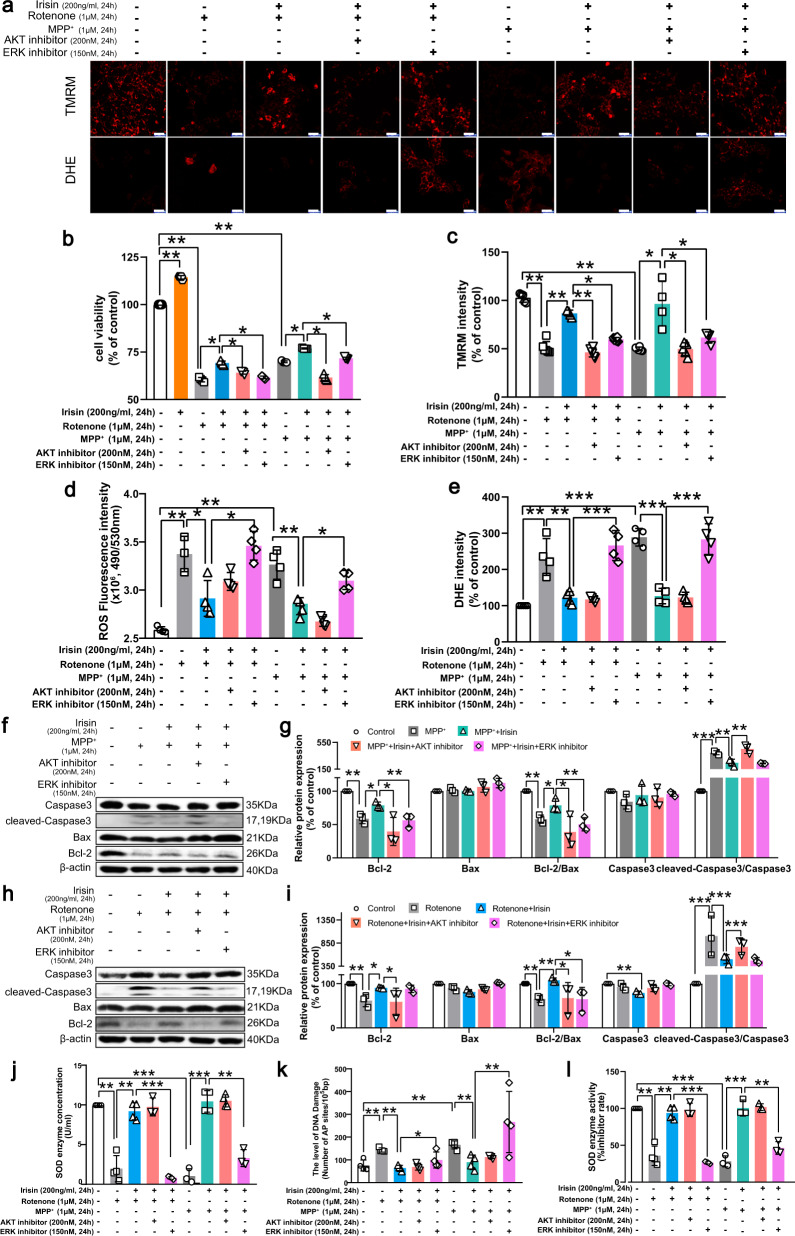
Irisin improves apoptosis and oxidative stress via activation of Akt and ERK1/2 signaling pathways in PD models induced by neurotoxins. SH-SY5Y cells were seeded and incubated until 80% cell density. The cells were separately treated with vehicle, rotenone (1 μM, 24 h), MPP^+^ (1 μM, 24 h), the co-treatment of rotenone (1 μM, 24 h) and irisin (200 ng/ml, 24 h), the co-treatment of MPP^+^ (1 μM, 24 h) and irisin (200 ng/ml, 24 h), the co-treatment of Akt inhibitor (200 nM, 24 h) and rotenone (1 μM, 24 h) and irisin (200 ng/ml, 24 h), the co-treatment of ERK1/2 inhibitor (150 nM, 24 h) and rotenone (1 μM, 24 h) and irisin (200 ng/ml, 24 h), the co-treatment of AKT inhibitor (200 nM, 24 h) and MPP^+^ (1 μM, 24 h) and irisin (200 ng/ml, 24 h), the co-treatment of ERK1/2 inhibitor (150 nM, 24 h) and MPP^+^ (1 μM, 24 h) and irisin (200 ng/ml, 24 h). **a**, **c**, **e** Confocal image and relative quantitative analysis of cell coverslips stained by TMRM and DHE staining and its quantification showing MMP level and ROS level in various groups of in vitro experiments. **b** CCK8 assay showing cell viability in various groups of in vitro experiments. **d** DCFH-DA assay showing ROS level in various groups of in vitro experiments. **f**, **g** Western blot showing the quantification of cleaved-caspase3/caspase3 ratio, Bax, Bcl-2, and Bcl-2/Bax ratio in each group of SH-SY5Y cells treated by irisin, MPP^+^, Akt inhibitor and ERK1/2 inhibitor. **h**, **i** Western blot showing the quantification of cleaved-caspase3/caspase3 ratio, Bax, Bcl-2, and Bcl-2/Bax ratio in each group of SH-SY5Y cells treated by irisin, rotenone, Akt inhibitor and ERK1/2 inhibitor. **j**, **l** SOD enzyme activity and SOD enzyme concentration in various groups of in vitro experiments. **k** The level of AP sites in various groups of in vitro experiments. All data are presented as mean ± SEM (*n* ≥ 3). ^∗^*p* < 0.05, ^∗∗^*p* < 0.01, and ^∗∗∗^*p* < 0.001.

To explore the roles of these pathways, we utilized Akt inhibitor and ERK inhibitor to block irisin-induced pathway activation and selected suitable doses of inhibitors, which effectively inhibited irisin-induced recovery of Akt and ERK1/2 pathways in PD models induced by neurotoxins (Supplementary Fig. 4e–h) but did not disturb the cell viability and ROS level of SH-SY5Y cells (Supplementary Fig. 4a–d). As a result, Akt inhibitor and ERK inhibitor hindered irisin-induced inhibition of apoptosis in PD models induced by neurotoxins, which was reflected by the expression levels of MMP, Bcl-2, cleavage of casepase 3, and the ratio of Bcl-2/Bax (Fig. 8a–e, f–i). For oxidative stress, irisin-induced ROS suppression, DNA damage renovation and SOD activation were significantly thwarted by ERK inhibitor (Fig. 8d, j–l). Thus, irisin prevented apoptosis inducing by MPTP, MPP^+^ or rotenone via Akt and ERK signaling pathways, and suppressed oxidative stress via ERK signaling pathway in PD models induced by neurotoxins.

### Irisin refreshes mitochondrial function via activation of Akt and ERK1/2 signaling pathways in PD models induced by neurotoxins

**Fig. 9:**
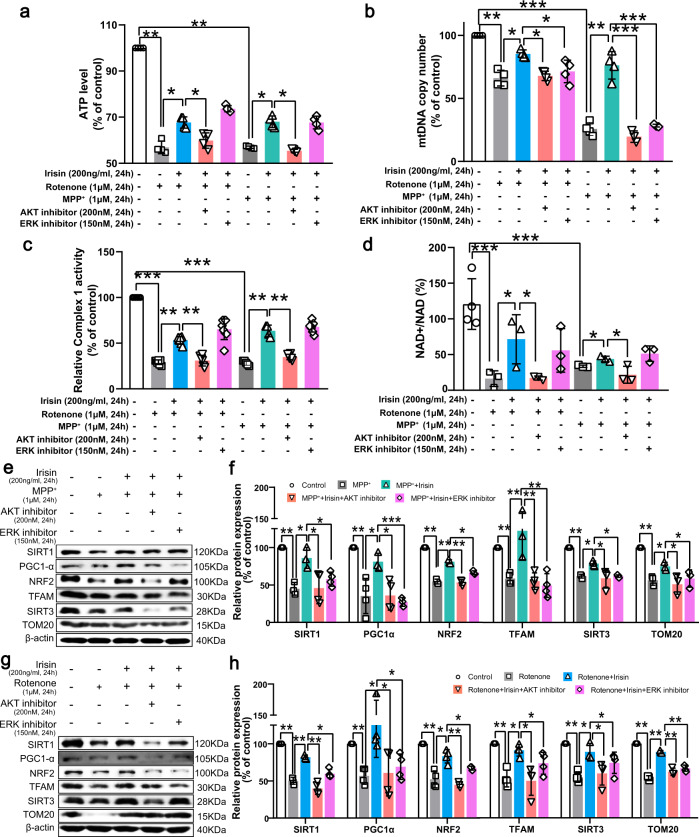
Irisin renews mitochondrial function via activation of Akt and ERK1/2 signaling pathways in PD models induced by neurotoxins. **a** The ATP level in various groups of in vitro experiments. **b** mtDNA copy number in various groups of in vitro experiments. **c** Mitochondrial complex I activity in various groups of in vitro experiments. **d** NAD^+^/NADH ratio in various groups of in vitro experiments. **e**, **f** Western blot showing the quantification of SIRT1, PGC-1α, NRF-2, SIRT3, TFAM and TOM20 in each group of SH-SY5Y cells treated by irisin, MPP^+^, Akt inhibitor and ERK1/2 inhibitor. **g**, **h** Western blot showing the quantification of SIRT1, PGC-1α, NRF-2, SIRT3, TFAM and TOM20 in each group of SH-SY5Y cells treated by irisin, rotenone, Akt inhibitor and ERK1/2 inhibitor. All data are presented as mean ± SEM (*n* ≥ 3). ^∗^*p* < 0.05, ^∗∗^*p* < 0.01, and ^∗∗∗^*p* < 0.001.

To test whether Akt and ERK1/2 signaling pathway were sufficient to obstruct the irisin-caused reconstruction in mitochondrial function in PD models induced by neurotoxins, we evaluated mitochondrial function after co-treatment of neurotoxin, irisin and these two inhibitors. Consequently, Akt inhibitor could reduce the irisin-induced enhancement of NAD^+^/NADH ratio, ATP level and complex I activity (Fig. 9a–d). For mitochondrial biogenesis, Akt inhibitor and ERK inhibitor impeded the irisin-produced restoration of SIRT1, PGC-1α, NRF-2 and TFAM expression, and prevented the restoration of TOM20 expression (Fig. 9e–h). Additionally, the recovery of irisin on mtDNA copy number was hampered due to the use of Akt inhibitor and ERK inhibitor in PD models (Fig. 9b). Hence, irisin refreshed mitochondrial function via of Akt and ERK1/2 signaling pathways in PD models induced by neurotoxins.

### Irisin restores mitochondrial morphology via activation of Akt and ERK1/2 signaling pathways in PD models induced by neurotoxins

**Fig. 10:**
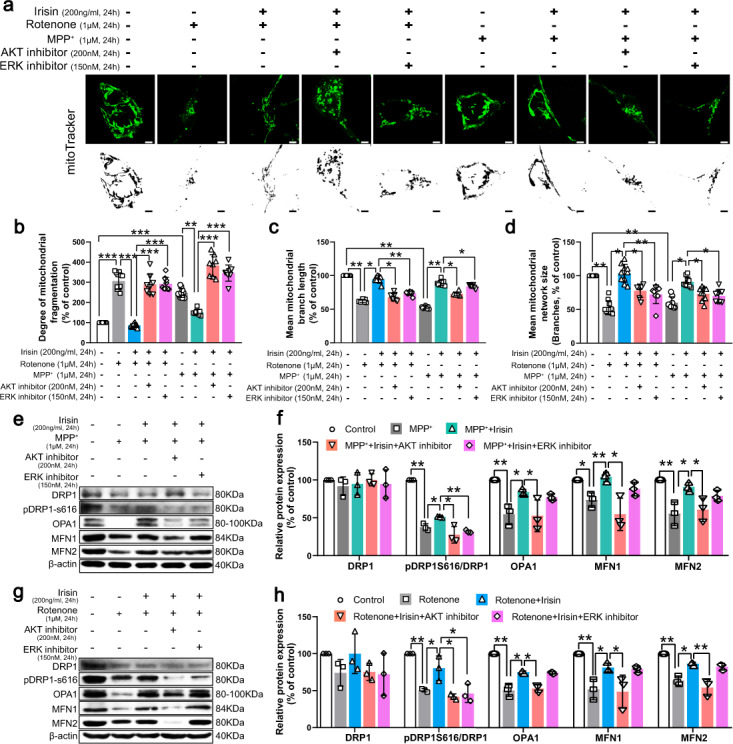
Irisin restores mitochondrial morphology via activation of Akt and ERK1/2 signaling pathways in PD models induced by neurotoxins. **a**–**d** Confocal image and relative quantitative analysis of cell coverslips stained by mitoTracker and its quantification showing mitochondrial fragmentation in various groups of in vitro experiments. Scale bar, 2 μm. **e**, **f** Western blot showing the quantification of DRP1, pDRP1-S616/DRP1 ratio, OPA1, MFN1and MFN2 in each group of SH-SY5Y cells treated by irisin, MPP^+^, Akt inhibitor and ERK1/2 inhibitor. **g**, **h** Western blot showing the quantification of DRP1, pDRP1-S616/DRP1 ratio, OPA1, MFN1and MFN2 in each group of SH-SY5Y cells treated by irisin, rotenone, Akt inhibitor and ERK1/2 inhibitor. All data are presented as mean ± SEM (*n* ≥ 3). ^∗^*p* < 0.05, ^∗∗^*p* < 0.01, and ^∗∗∗^*p* < 0.001.

To address how irisin regulate mitochondrial morphology, we further assessed whether irisin could stabilize mitochondrial morphology through Akt and ERK pathway in PD models induced by neurotoxins. According to mitoTracker results, the effect of irisin on decreasing mitochondrial fragmentation was abolished by Akt and ERK inhibitors (Fig. 10a–d). Due to the treatment of Akt inhibitor, molecules that regulate mitochondrial fusion, including OPA1, MFN1 and MFN2, remained in low expression levels in irisin-treated PD models (Fig. 10e–h). And, phosphorylation of DRP1 at Ser616 regulating by irisin to increase mitochondrial fission could maintain the damaged state in irisin-treated PD models because of the use of Akt and ERK inhibitor (Fig. 10e–h). In a word, irisin restores mitochondrial morphology via activation of Akt and ERK1/2 signaling pathways in PD models induced by neurotoxins.

## Discussion

In the present study, we showed that administration of exogenous irisin alleviated neuronal injury in PD models induced by neurotoxins. The potential mechanisms might be explained by the effect of irisin on improving mitochondrial function, promoting mitochondrial biogenesis and ATP synthesis, and alleviating oxidative stress. And exogenous irisin played a vital role in protecting mitochondria through Akt and ERK1/2 signaling pathway. Briefly, irisin may be a promising candidate for preventing or treating PD.

Firstly, we discovered that serum irisin levels in PD patients increased after regular exercise, and the increased levels of irisin had a positive correlation with the increase of BBS scores. More specifically, the exercise-induced increase in serum irisin levels was associated with ameliorated balance function which could reduce the risk of falls and postural instability, and improve the living quality of PD patients. Furthermore, elevation of circulating irisin levels by peripheral delivery (i.p.) of exogenous irisin could improve motor dysfunction and pathological injury of MPTP treated mice. The expression levels of BDNF in brains of MPTP treated mice with irisin pre-treatment was increased, but there was no change in MPTP treated mice with irisin delayed treatment. These results indicated that BDNF might not be the vital target by which irisin prevented neuronal damage in MPTP treated mice. In addition to TH-positive dopaminergic cells, we also detected and analyzed TUJ1-positive neurons, GFAP-positive astrocytes, OLIGO2-positive oligodendrocytes and IBA1-positive microglia in the midbrains of mice in each group. Remarkably, irisin could significantly reduce the activated IBA1-positive microglia in MPTP treated mice, suggesting that irisin could improve the inflammation in the brain of MPTP treated mice^29,48^. Actually, neuroinflammation is a well-established feature of PD and aggregated α-syn can induce microglia activation and inflammatory cytokine release much earlier than the occurrence of DA cell death. And reactive microglia could accelerate α-syn transmission to induce neurodegeneration^49,50^.

Mitochondrial dysfunction is a feature observed in both sporadic and monogenic PD^51,52^. Based on this view, classical animal models of PD induced by neurotoxins (MPTP, rotenone, and so on) that cause mitochondrial dysfunction have been developed. We also chose these models in our present study. These neurotoxins facilitate caspase activation leading to mitochondrial dysfunction, and activate cell apoptosis process. Here, we demonstrated that irisin inhibited mitochondria-dependent apoptosis induced by neurotoxins in models through increasing Bcl-2 level, and decreasing MMP level, as well as caspase-3 cleavage and activation.

One of the remarkable findings in the present study is that both pretreatment or treatment with exogenous irisin could increase mitochondrial complex I activity and promote mitochondrial biogenesis in PD models. Mitochondrial complex I to IV form the respiratory chain, which facilitates electron transfer and drives ATP synthesis^37,38,53^. Except complex II subunits that are all encoded by the nuclear genome, the remaining enzymes contain subunits of dual genetic origin, nuclear DNA-encoded subunits and mtDNA-encoded subunits^54^. Hence, impaired mtDNA copy number impacts biogenesis and function of mitochondrial complexes. Complex I dysfunctions are attributed to decreased catalytic activity and/or increased production of ROS^34,55^. When complex I is damaged, the mitochondrial NAD^+^/NADH ratio reduces. The use of irisin in PD models could improve mitochondrial complex I activity and mtDNA copy number, promote NAD^+^ regeneration and ATP synthesis^56^. Then the function of NAD^+^-dependent histone/protein deacetylases (sirtuins) is restored by concomitant increases in NAD^+^, like SIRT1. SIRT1 activates PGC-1α-mediated transcription of nuclear and mitochondrial genes encoding (TFAM, NRF1, NRF2) for proteins promoting mitochondria proliferation, oxidative phosphorylation and energy production^57–60^. And SIRT3 as a downstream target gene of PGC-1α mediates intracellular ROS production and stimulates mitochondrial biogenesis^61,62^.

Superoxide production by mitochondrial complex I is governed by NAD^+^/NADH ratio^56^. Under normal conditions, NAD^+^/NADH is high and superoxide production is low, but when NAD^+^/NADH reduces, superoxide production increases. A vicious cycle occurs in which oxidative damage of mitochondrial complex I decreases its activity, further stimulating superoxide production^63^. Our study found that irisin significantly decreased ROS level by regulating mitochondrial dysfunction and improving impaired anti-oxidant system, especially SOD activity. Furthermore, induced ROS accumulation could react with DNA to produce a myriad of cytotoxic and mutagenic base damage, like 8-oxoG and AP site. And we showed that irisin treatment not only avoided ROS-induced AP site, but also restored OGG1 that is the major enzyme for repairing 8-oxoG.

Mitochondria constantly undergo the balance of fusion and fission to maintain mitochondrial homeostasis^40,41^. The mitochondrial fusion in inner mitochondrial membrane is regulated by OPA1, whereas MFN1 and MFN2 is required for outer mitochondrial membrane fusion. Mitochondrial fission is controlled by protein level and phosphorylation modification of DRP1^64^. Phosphorylation of DRP1 at Ser616 increases mitochondrial fission, whereas phosphorylation at Ser637 reduces mitochondrial fission. Damaged mitochondria might be restored by the fusion with neighboring intact mitochondria via preserving a homogenous mitochondrial population and diluting DNA faults^65,66^. And mitochondrial fission also plays a necessary role in cell survival, by removing dysfunctional mitochondria, thus alleviating ROS accumulation and oxidative damage^67^.

Notably, we confirmed that irisin improved mitochondrial function through Akt and ERK1/2 signaling pathway in the present study. It is reported that irisin mediates its effects via αV integrin receptors and following FAK/Akt signaling pathway, whereas exogenous irisin could also target mitochondria in A549 cells and myocardial cell^17,27,28^. Hence, we further explored the way irisin exerted its effect. From our data, irisin could not be transported into the cells directly whereas it could bind to its receptor on membrane to activate downstream reactions.

Although our data identified peripheral irisin delivery as an effective approach to alleviate motor symptoms in PD, it remained undetermined whether benefits with exercise were irisin-dependent. Of note, our PD models based on neurotoxin could not accumulate large amounts of α-synuclein shortly, therefore whether irisin could specifically target the formation or removal of α-synuclein could not be determined. Furthermore, only retrospective data of PD patients provided in our study, prospective trials should be launched to further examine the effect of irisin on PD patients. Anyway, there is no denying the fact that irisin treatment was effective in PD models, which shed light on its application into human disease. In conclusion, peripherally delivered irisin appears to be a promising therapeutic approach for PD.

## Methods

### Study population

In this study, we included 23 patients with PD, who accepted exercise classes from June 2014 to December 2015 in Shanghai Tongji Hospital, Tongji University School of Medicine. The inclusion criteria were as follows: (i) recruited patients of PD were evaluated by neurologists using the United Kingdom Parkinson’s Disease Society Brain Bank clinical diagnostic criteria; (ii) patients were asked to follow the original medication dosage during the entire study; (iii) patients accomplished exercise twice a week for 12 weeks as described in the previous study;^68^ (iv) the effect of exercise on PD was assessed by Parkinson’s Disease Rating Scale Motor Examination (UPDRSIII), and the Berg Balance Scale (BBS); (v) assessment was completed by clinicians who were blinded to the mode of exercise intervention before and at the end of the 12th weeks; (vi) blood samples of patients in fasting state were collected by a standard procedure for RNA isolation before the start and 48–72 h after the completion of the 12-week training program, and serum samples that was isolated immediately were preserved for long-term storage at −80 °C^68^. The exclusion criteria were that patients had other medical conditions. Finally, the population included in the study were all from the clinical trial that was approved by the Ethics Committee of Tongji Hospital, performed in accordance with the Declaration of Helsinki, and was registered in the Chinese Clinical Trial Registry (ChiCTR-TRC-14004707, http://www.chictr.org.cn/showproj.aspx?proj=4866). Written informed consent was obtained from each participant.

### Animal model and tissue preparation

Male C57BL/6 mice were purchased from Shanghai SLAC Laboratory Animal Company. All animal experiments were performed in accordance with the National Institutes of Health Guide for the Care and Use of Laboratory Animals and were approved by the ethics committee for the use of experimental animals in Tongji University. Male mice (6–8 weeks old, weighing 20–30 g, 6–10 per group) were injected a daily intraperitoneal injection (i.p.) of MPTP (Sigma–Adrich, USA, M0896; 30 mg/kg weight), irisin (R&D, China, 8880-IR; 200 μg/kg weight), or vehicle treatment. The detailed treatment plans were showed in Fig. 1a, l. After behavioral tests and euthanasia, brain tissues of these mice were immediately collected, dehydrated by sucrose gradient and fixed by 4% paraformaldehyde, and were transferred to the optimal cutting temperature for long-term storage at −80 °C. According to the mouse brain atlas, striatal brain tissue between Bregma 1.25 mm and Bregma −1.00 mm was selected for serial section. The mesencephalic tissue between Bregma −3 mm and Bregma −4.75 mm was selected for serial section. The coronal section was sectioned as 10 µm sections on a cryostat (Leica CM3050) and kept on polylysine-coated slides at −80 °C. About 170 frozen brain sections were obtained from each mesencephalic tissue. For TH staining, one frozen brain section was taken every five frozen brain sections for stratified sampling. The complete unilateral substantia nigra region was visualized and captured under 10X eyepiece and 20X objective. Under this condition, TH (+) and Nissl (+) cells could be counted in the whole substantia nigra area. Instead of area sampling, the whole region was regarded as the region of interest. A total of 32–34 frozen brain sections were observed and counted for each mouse. Quantitative analysis was carried out according to the following formula: *N* = ∑Q / (h ∙ a ∙ ∑P), where N is TH (+) and Nissl (+) cell density (cells/mm^3^), Q is the number of TH positive cells in a region of interest, h is thickness of frozen brain sections (10 µm), a is the area of the region of interest and P is the number of the region of interest^69,70^. The mouse brains intended for cell lysis (3–4 per group) were transcardially perfused with ice-cold PBS, transferred to liquid nitrogen for long-term storage at −80 °C and further experiments.

### Behavioral tests

Motor functions were tested with rotarod tests and pole tests. These tests were performed according to previously published methods^71^. In rotarod tests, mice were trained at the speed of 4 RPM for 2 min, and then the Rota Rod was accelerated uniformly from 4 RPM to 40 RPM for the longest time of 4 min. The time and speed of the mice falling from the rotary bar were recorded. In pole tests, a wooden pole with a height of 50 cm and a diameter of 0.5 cm was used, which was wrapped with gauze to prevent skidding. Under the wooden pole was a base to keep balance, and there was a wooden ball on the top, about the size of a ping-pong ball. Mice were placed on a wooden pole and learned to crawl down. The total time for mice to climb from the top to the bottom of the wooden pole was recorded. The experiment was carried out three times independently, and the average value of the three times was taken as the experimental result.

### Cell culture and drug treatment

Human neuroblastoma SH-SY5Y cells were cultured in Dulbecco’s Modified Eagle’s Medium (Hyclone, USA) mixed with 10% fetal bovine serum (FBS, Gibco, USA) at 37 °C in a humidified incubator (Thermo Fisher Scientific, Wilmington, MA, USA) supplied with 5% CO_2_. Rotenone (Sigma–Adrich, USA, R8875) and 1-methyl-4-phenylpyridinium (MPP^+^; Sigma–Adrich, USA, D048) were dissolved in dimethyl sulfoxide (DMSO, Sigma–Adrich, USA, D2650, final concentration was 0.01%). These two toxins are widely used in PD researches because they can mimic certain pathological phenomena in PD, such as apoptosis, oxidative stress and mitochondrial dysfunction, etc^72,73^. Akt Inhibitor VIII, Isozyme-Selective, Akti-1/2 (200 nM, 24 h, CAS 612847-09-3, Santa Cruz), ERK Inhibitor II, FR180204 (150 nM, 24 h, CAS 865362-74-9, Santa Cruz), iRGD peptide (300 nM, 24 h, HY-PO122, MCE, USA), irisin (R&D, China, 8880-IR) and his-tagged irisin (his-irisin, Novoprotein, China, CM35) was dissolved in sterile PBS. Cells were treated with rotenone at the indicated time points and doses, as well as MPP^+^ and irisin (Supplementary Fig. 2a–e). Finally, the duration and time of reagents were determined (Rotenone: 1 μM, 24 h; MPP^+^: 1 μM, 24 h; and irisin: 200 ng//mL, 24 h). After being digested and washed with phosphate buffer saline (PBS), SH-SY5Y cells were subjected to further treatment and analysis.

### Enzyme-linked immunosorbent assay (ELISA)

Irisin level of human serum was detected using the corresponding kits (BioVision, USA, K4761) as follows: (1) 50 μL different standards and 50 μL diluted serum were in duplicate added into the 16-well strips that coated with irisin protein; (2) 50 μL Detection Antibody was added into each well, and the mixture was incubated for 1 h at 37 °C; (3) Wash Buffer was used to wash the strips and 100 μL diluted HRP Conjugated anti-rabbit IgG (HRP) was then added into each well to incubate for 1 h at 37 °C; (4) after washing, 100 μL TMB substrate solution was added into each well for the color reaction at room temperature in the dark for 20 min, and 100 μl Stop Solution was added to stop the reaction; (5) the Optical Density (OD) at 450 nm was measured in automated microplate reader (SpectraMax iD3).

### Immunofluorescent (IF) staining

Cultured cells on coverslips and frozen brain sections for IF were fixed with 4% paraformaldehyde in PBS for 15 min, washed with PBS three times, and then permeabilized in 0.1–0.3% Triton X-100 for 30–60 min or not. Then these sections were sequentially blocked with donkey serum (3%, 1 h), incubated with primary antibody (4 °C, 8 h), and incubated with secondary antibody at room temperature (26 °C, 1 h) and sealed by HistoMount (ThermoFisher, USA, 008030). Images were captured by fluorescence microscopy (OLYMPUS BX53) or confocal microscopy (Leica SP8). The mean intensity of fluorescence was measured using Image J software.

### Transmission electron microscopy (TEM)

For TEM morphological analysis, freshly cell pellets were fixed in 2.5% glutaraldehyde and FBS at 4 °C for 12 h. After being washed with phosphate Buffer three times, they were fixed with 1% osmium tetroxide for 2 h and dehydrated through a graded ethanol series at 4 °C. Finally, the samples were embedded in epoxy resin at 60 °C for 2 h. Ultrathin sections (70 nm) were assembled to a copper grid and observed using electron microscopy (Hitachi, Japan).

### Western blot analysis

Cultured cells and brain tissues were lysed with RIPA lysis buffer (Beyotime, China, P0013B) supplemented with protease inhibitor cocktail (Roche, Switzerland, 4693116001) and phosphatase inhibitor cocktail (Roche, Switzerland, 4906845001). BCA assay kits (Beyotime, China, P0010) were used to measure total protein concentrations. Then western blot analysis was performed according to previously published methods^71^. Related antibodies are listed in Supplementary Table 1. After being washed in 0.1% TBST three times, protein bands were activated by ECL Western Blotting Substrate kit (Merck Millipore, Alemanha, WBLUF0500) and protein signals were tested on the ImageQuant LAS 4000mini system. ImageJ software was applied for analyzing band density.

### Measurement of reactive oxygen species (ROS) level and cell viability

Briefly, cells were seeded in a 96-well microplate (1 × 10^4^ cells/well) and were cultured for 24 h. After treated with rotenone, MPP^+^ and irisin as previously described, the medium was replaced by CCK8 (DOJINDO, China, CK04, diluted 10 times in medium to use) or DCFH-DA (Jiancheng, China, E004, final concentration 10μm), followed by incubation at 37 °C for 4 h. Then the CCK8 level and DCFH-DA level were detected by automated microplate reader (SpectraMax iD3).

### Measurement of DNA damage abasic site (AP site), NAD^+^/NADH ratio, malondialdehyde (MDA) and superoxide dismutase (SOD) activity

After being treated with the indicated drugs, the cells were harvested, suspended in PBS and then fragmented with ultrasonic liquid processors (Sonicator4000). We tested AP site (DOJINDO, China, DK02) as follows: (1) the cell total DNA solution was isolated as template using DNeasy Blood and Tissue Kit (Qiagen, CA, 69504); (2) 10 μL DNA solution and 10 μL ARP solution were added into a tube to incubate at 37 °C for 1 h, and the ARP-labeled DNA was purified with a Filtration Tube; (3) 90 μL ARP-labeled DNA solution and 310 μL TE were added into a tube, then 60 μL the diluted solution or ARP-DNA Standard Solution were added into each well on 96-well plate, after which 100 μL DNA Binding Solution was added into each well for incubation at room temperature overnight; (4) after discarding the solution and washing the well, 150 μL diluted HRP-Streptavidin solution was added into each well to incubate at 37°C for 1 h; (5) after discarding the solution and washing the well, 100 μL Substrate Solution was added into each well to incubate at 37°C for 1 h; (6) the OD at 650 nm was measured on automated microplate reader (SpectraMax iD3), and the number of AP sites in the sample DNA were determined using a calibration curve. For NAD^+^/NADH ratio (BioVision, USA, K337-100), the levels of both NADt (total NAD^+^ and NADH) and NADH can be easily measured; the level of NAD^+^ can be easily calculated by subtracting NADH from NADt. For NADH measurement, samples were heated to 60 °C for 30 min to decompose NAD^+^ , and were cooled down on ice (this step is not necessary for measurement of total NAD^+^ /NADH). Then samples and standards were added into wells, and reaction mix was added into each well for 5-min incubation at room temp to convert NAD^+^ to NADH. NADH developer was added into each well to incubate for 4 h. Then the OD at 450 nm was measured in automated microplate reader (SpectraMax iD3). For MDA level (BioVision, USA, K739-100), 50 µL MDA standards or samples were added into wells of MDA Conjugate coated plate and were incubated for 10 min. 50 µL the diluted anti-MDA antibody was added into each well to incubate for 1 h. After washing, 100 µL diluted Secondary Antibody-HRP Conjugate was added into each well to incubate for 1 h. After washing again, 100 µL warm Substrate Solution was added into each well to incubate for 20 min, then 100 µL Stop Solution were added into each well to stop the enzyme reaction. The OD at 450 nm was measured immediately on automated microplate reader (SpectraMax iD3). For SOD activity (DOJINDO, China, S311), 20 μL standards, ultra-pure water or sample solution were respectively added into standard wells, control wells, or sample wells. Then 200 μL WST working solution was added into each well, after which 20 μL enzyme working solution was added into each well to incubate at 37 °C for 20 min. The OD at 450 nm was measured and determine SOD activity using a calibration curve.

### Measurement of Complex I activity and ATP levels

The cells in 96-well plate were firstly treated with the indicated drugs. For Complex I activity measurement (Abcam, USA, ab109721), detergent was initially added into each well to extract prepared samples loaded onto plate and to incubate for 3 h at room temperature. Then the plate wells were washed with Buffer, after which 200 µL Assay Solution was added to each well. We measured OD450 nm in kinetic mode by automated microplate reader (SpectraMax iD3) at room temperature for up to 30 min. For ATP levels measurement (Abcam, USA, ab113849), ATP standards were added into standard wells and media were added into control wells in the same plate containing cells to be analyzed. Detergent solution was then added into each well for 5-min incubation for cells lysis and ATP stabilization. And substrate solution was added into each well for 10-min incubation in darkness, and the luminescence was measured by automated microplate reader (SpectraMax iD3).

### Transferase-mediated deoxyuridine triphosphate-biotin nick end labeling (TUNEL) staining

After treatment, cells cultured on cover slips in 24-well dishes were fixed with 4% paraformaldehyde for 30 min, then washed three times with PBS before treatment with 0.1% Triton X-100 for 10 min for permeabilization. After washed, cells were incubated with the TUNEL reaction mixture as described in manufacturer’s instructions (TUNEL BrightRed Apoptosis Detection Kit, Vazyme Biotech Co., Ltd, A113), and nuclei were then stained with DAPI for 5 min. Images were captured by fluorescence microscopy (OLYMPUS BX53). The images were measured using Image J software.

### Dihydroethidium (DHE) staining

Frozen brain sections were washed with PBS three times and laid out to dry. Cultured cells in confocal dishes were also washed with PBS three times. Cells and tissues were incubated with DHE (5μm, Sigma, USA, D7008) at 37 °C for 30 min, then were washed three times. Images were captured by fluorescence microscopy (OLYMPUS BX53) or confocal microscopy (Leica SP8). The mean intensity of fluorescence was measured using Image J software.

### Measurement of mitochondrial membrane potential (MMP) and mitochondrial morphology

Mitochondrial membrane potential and mitochondrial morphology were evaluated by TMRM (Thermo Fisher, USA, T5428) and mitoTracker Green FM (Thermo Fisher, USA, M7514). After treatment, cells cultured in confocal dishes were incubated with TMRM (diluted 1000 times in medium to use) or mitoTracker Green (diluted to 200 nM in medium to use) at 37 °C in dark place for 30 min, then were washed three times with PBS. Images were captured by confocal microscopy (Leica SP8), and were analyzed using Image J software. Mitochondrial morphology was analyzed by MiNA plug-in in Image J software^74^, which could skeletonize and transform the mitochondrial network fluorescence images for structural analysis, including the number of mitochondria, the number of mitochondrial networks, the number of mitochondrial network branches and the average length. The results included Mitochondrial Individuals (Counts), Mitochondrial Networks (Counts), Mean Branch Length (Inches), Mean Network Size (Branches, Counts) and Network Size Standard Deviation. After the results were summarized, the measurement data were analyzed and processed (the experiment was repeated for 3 times in each group, 2-3 dishes each time, and 8-10 cells were observed in each dish).

### Analysis of mtDNA copy number

The cell total DNA was isolated as template using DNeasy Blood and Tissue Kit (Qiagen, CA, 69504) and were analyzed by quantitative polymerase chain reaction (qPCR) using AceQ qPCR SYBR Green Master Mix (Vazyme Biotech Co., Ltd, Q111). mtDNA copy number was determined as the copy number of mtDNA encoded mtND1 and the normalized contrast was determined as the copy number of the nuclear encoded NDUFV1 gene. The proteins encoded by mtND1 and NDUFV1 are subunits of mitochondrial complex I. The primers were synthesized by GENEWIZ (Nanjing, China) as follow: mtND1: Forward 5'-TCCTGCCATCATGACCCTTG-3', Reverse 5'- CTGCGGCGTATTCGATGTTG -3'; NDUFV1: Forward 5'- TAGGAGCTTCACCGTGGGAG -3', Reverse 5'- TTGCCAATCAGACCTGCCTC -3'. The relative levels were calculated by the Comparative-Ct Method (ΔΔCt).

### RNA-Sequencing and analysis

For bulk RNA sequencing, the fresh brain tissue of mice was collected after perfusing. Nigral tissues were manually extracted and instantly froze in liquid nitrogen. All samples were subjected to RNA-seq by the LC Bio (Zhejiang, China) and data analysis. The libraries were sequenced on Illumina NovaSeq 6000. Gene read counts were calculated with featureCounts. Downstream analysis was performed within the R environment.

### Statistical analysis

All data are presented as mean ± standard deviation (SD). Each experiment was replicated at least three times. Data visualization and analysis were performed with GraphPad Prism 8 (GraphPad Software Inc., La Jolla, CA, USA) and SPSS 20.0 (SPSS Inc., Chicago, Illinois, USA). Statistical analysis was performed using a two-tailed paired *t*-test, a two-tailed student’s *t*-test or a two-tailed one-way ANOVA. For the data that meet homoscedasticity or meet homoscedasticity after Log change, one-way ANOVA was used for comparison of population means, and Tukey test was used for multiple comparison. For the data that could not meet homoscedasticity, Dunnett T3 test was used for multiple comparison. Significant difference was assessed as ns *p* > 0.05, ^∗^*p* < 0.05, ^∗∗^*p* < 0.01, and ^∗∗∗^*p* < 0.001.

### Reporting summary

Further information on research design is available in the Nature Research Reporting Summary linked to this article.

## Supplementary information

Supplementary materials

Reporting Summary

## Data Availability

RNA-Seq datasets generated here are uploaded to NCBI Gene Expression Omnibus (GEO) database (GSE number: GSE214542). All blots were processed in parallel and derive from the same experiment. Extra data are available from the corresponding author upon request.

## Code availability

Codes are available from the corresponding author upon request.
