# Supplementary material for: Irisin exhibits neuroprotection by preventing mitochondrial damage in Parkinson’s disease
Source: NPJ Parkinsons Dis. 2023 Jan 31;9:13. doi: 10.1038/s41531-023-00453-9 (PMC9889817; doi:10.1038/s41531-023-00453-9)

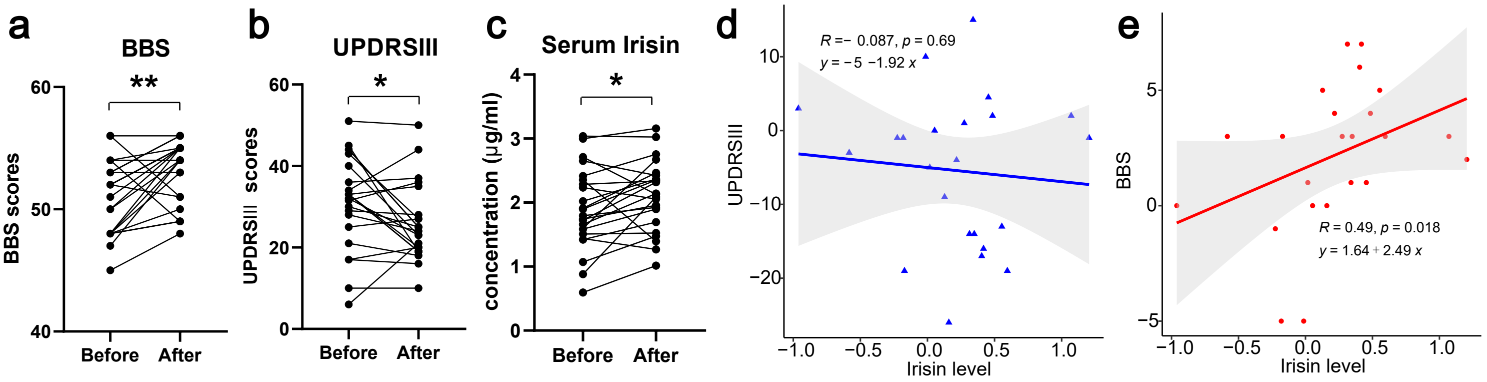

Supplementary Figure 1

The relationship between motor function of PD patients and their serum irisin concentration.

(**abc**) After 12 weeks of regular exercise, balance function scored by BBS (**a**), motor function evaluated by UPDRSIII (**b**) of PD patients were improved, and serum irisin concentration (**c**) of PD patients were increased. (**d**) The correlation between increased serum irisin concentration and improved motor function scored by UPDRSIII. (**e**) The correlation between increased serum irisin concentration and improved balance function scored by BBS. p values are determined by paired t-test and Pearson correlation coefficient. *p < 0.05, **p < 0.01, and ***p < 0.001 vs PD patients before 12-week regular exercise.

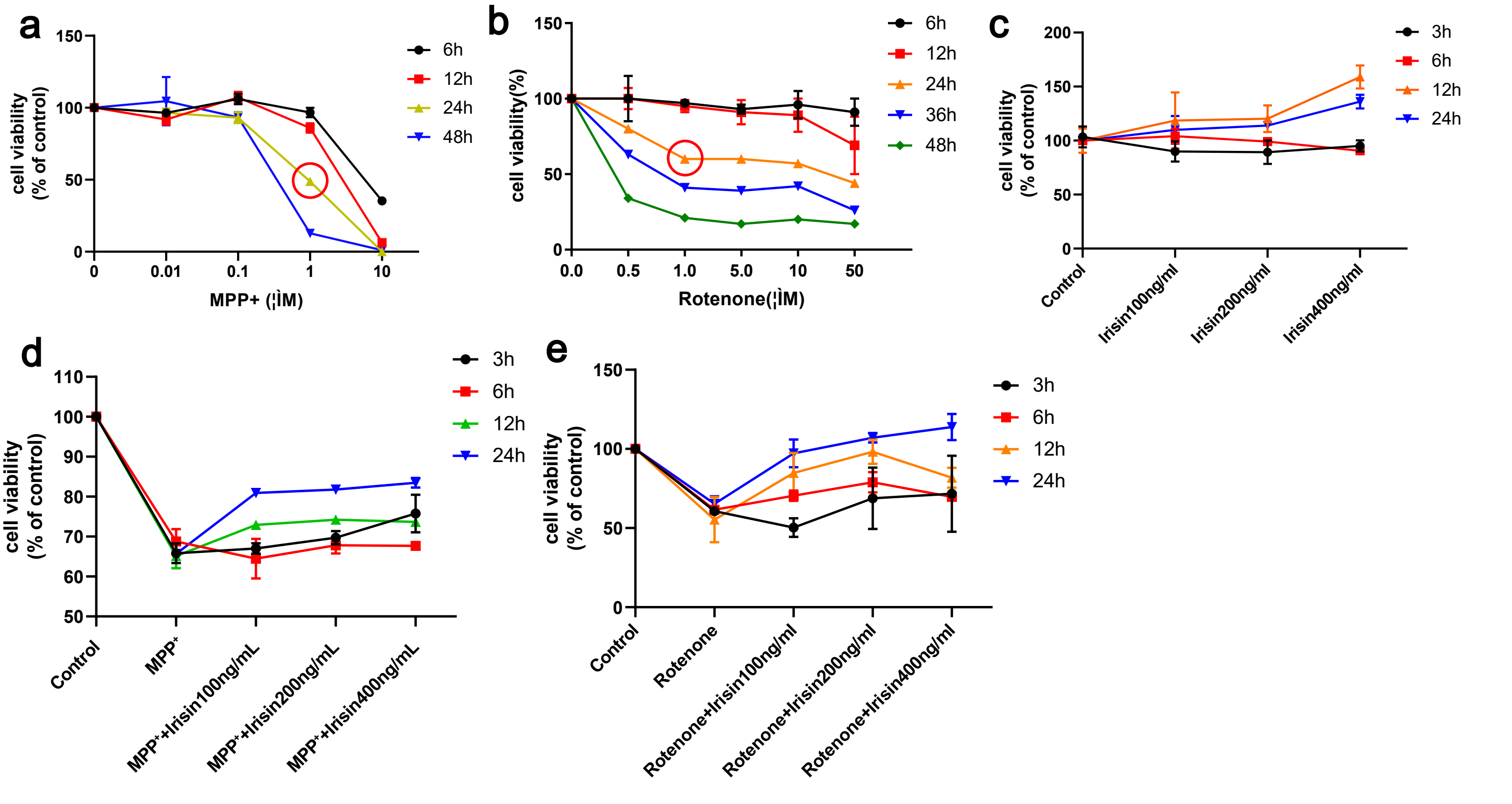

Supplementary Figure 2

The optimal dose of rotenone, irisin, MPP+ in SH-SY5Y cells.

SH-SY5Y cells were seeded and incubated until 80% cell density. (**abcde**) The cells were treated with indicated time and indicated concentration of rotenone, irisin, MPP+ for draw the time and does dependent curve of MPP+ (**a**), rotenone (**b**), irisin (**c**), irisin and MPP+ co-treatment (**d**), and irisin and rotenone co-treatment (**e**) in SH-SY5Y cells.

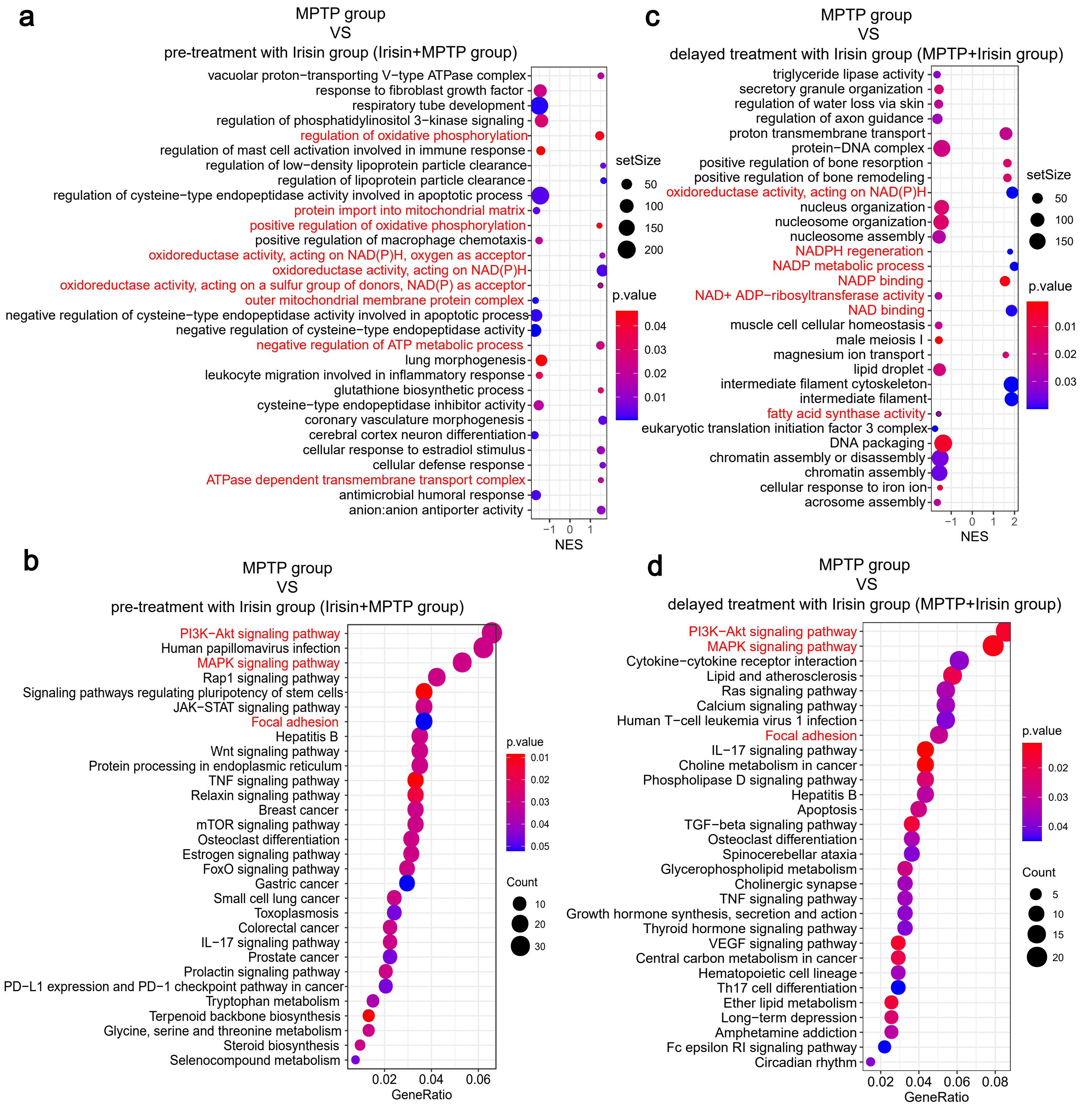

Supplementary Figure 3

The analysis of RNA-Seq in MPTP treated mice.

(**a**) GSEA analysis for dysregulated pathways in midbrain in MPTP treated mice receiving pre-treatment with irisin.

(**b**) KEGG analysis for dysregulated pathways in midbrain in MPTP treated mice receiving pre-treatment with irisin.

(**c**) GSEA analysis for dysregulated pathways in midbrain in MPTP treated mice receiving delayed treatment with irisin.

(**d**) KEGG analysis for dysregulated pathways in midbrain in MPTP treated mice receiving delayed treatment with irisin. KEGG, Kyoto Encyclopedia of Genes and Genomes.

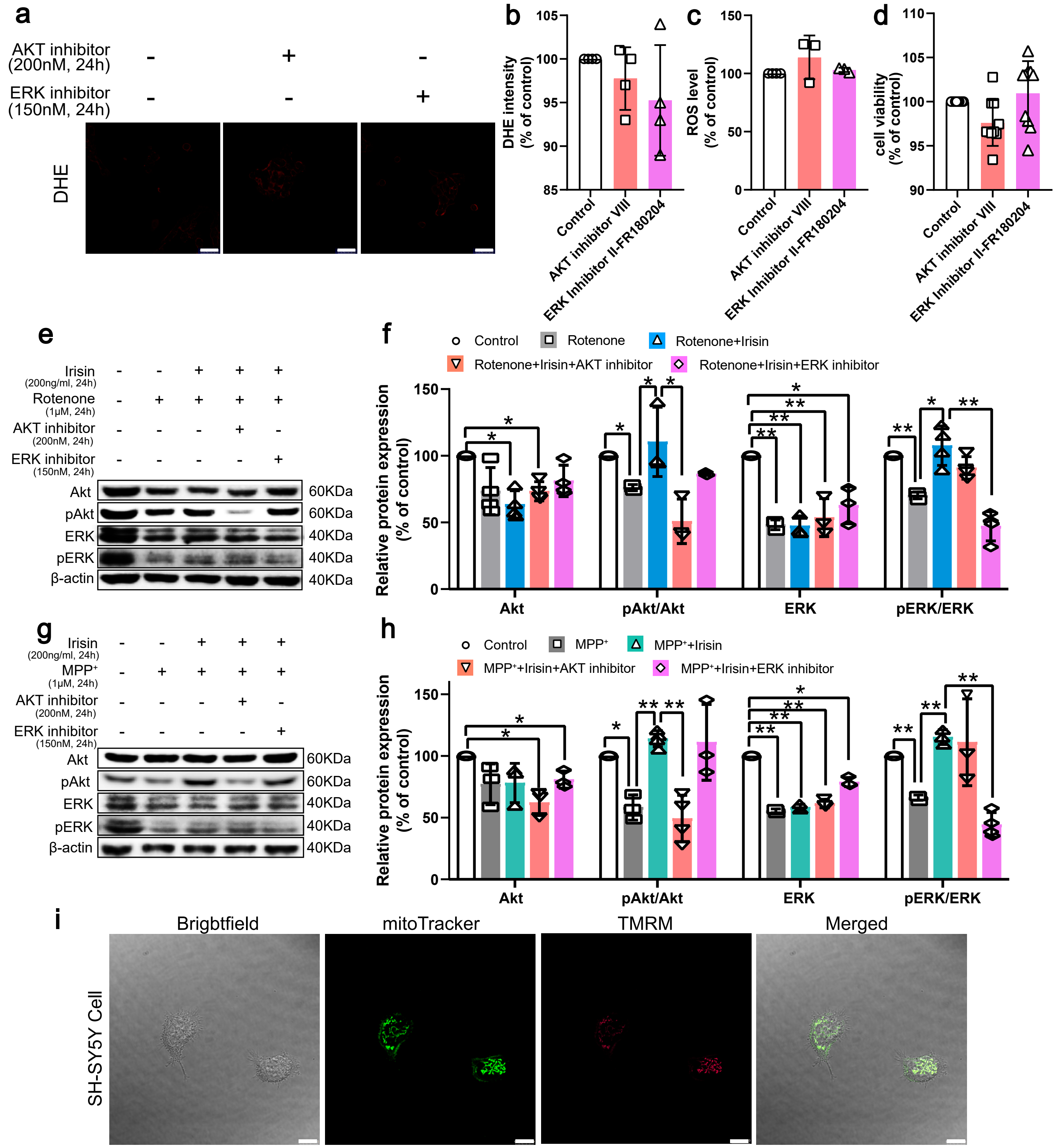

Supplementary Figure 4

The optimal dose of Akt inhibitor or ERK1/2 inhibitor.

The cells were treated with Akt inhibitor (200nM) or ERK1/2 inhibitor (150nM) for 24h to test its effect on cell viability (**d**) and ROS level detected by DHE staining (**ab**) and DCFH-DA assay (**c**). Without affecting cell viability, the specified concentration of Akt inhibitor (200nM) or ERK1/2 inhibitor (150nM) can effectively inhibit the corresponding signaling pathway in rotenone-induced (**ef**) or MPP+-induced (**gh**) PD models. (**i**) Confocal image of cell coverslips stained by mitoTracker and TMRM showing mitochondrial structure in SH-SY5Y cells. Scale bar, 2 μm. All data are presented as mean ± SEM (n = 3). p values are determined by one-way ANOVA. *p < 0.05, **p < 0.01, and *** < 0.001.

Supplementary Table 1. Antibodies used in immunofluorescence staining and western blot

Antibodies	Dilution	Species	Catalog	Company
TH	1:200(IF)/1:2500(WB)	Rabbit	AB152	Millipore
BDNF	1:2500(WB)	Rabbit	ab108319	Abcam
NeuroTrace Green Fluorescent Nissl Stains (500/525)	1:100(IF)	-	N-21480	ThermoFisher
Alexa Fluor Plus 555	1:1000(IF)	Goat	A32732	Invitrogen
HRP-conjugated anti-mouse IgG	1:10000(WB)	Goat	115-035-003	Jackson ImmunoResearch
HRP-conjugated anti-rabbit IgG	1:10000(WB)	Goat	111-035-003	Jackson ImmunoResearch
DRP1	1:2500(WB)	Rabbit	8570S	CST
phospho-DRP1(Ser616)	1:2500(WB)	Rabbit	AP0849	Abclonal
phospho-DRP1(Ser637)	1:2500(WB)	Rabbit	AP0812	Abclonal
OPA1	1:2500(WB)	Rabbit	80471S	CST
MFN2	1:2500(WB)	Rabbit	9482S	CST
UCP2	1:2500(WB)	Rabbit	89326S	CST
MFN1	1:2500(WB)	Rabbit	14739S	CST
SIRT1	1:2500(WB)	Rabbit	9475	CST
SIRT3	1:2500(WB)	Rabbit	5490	CST
PGC-1α	1:2500(WB)	Rabbit	ab54481	Abcam
TFAM	1:2500(WB)	Rabbit	ab131607	Abcam
NRF1	1:2500(WB)	Rabbit	69432	CST
NRF2	1:2500(WB)	Rabbit	12721S	CST
TOM20	1:2500(WB)	Rabbit	A19403	Abclonal
CASPASE3	1:2500(WB)	Rabbit	9662S	CST
cleaved-CASPASE3	1:2500(WB)	Rabbit	9661	CST
BAX	1:2500(WB)	Rabbit	A0207-100ul	Abclonal
BCL-2	1:2500(WB)	Rabbit	A0208-100ul	Abclonal
AKT	1:2500(WB)	Rabbit	4691	CST
phospho-AKT(Thr308)	1:2500(WB)	Rabbit	4056	CST
FAK	1:2500(WB)	Rabbit	3285	CST
phospho-FAK(Tyr397)	1:2500(WB)	Rabbit	3283	CST
ERK1/2	1:2500(WB)	Mouse	A10613	Abclonal
phospho-ERK1(T202/Y204) + ERK2(T185/Y187)	1:2500(WB)	Rabbit	AP0472	Abclonal
AMPKα1/2	1:2500(WB)	Mouse	sc-74461	Santa Cruz
phospho-AMPKα (Thr172)	1:2500(WB)	Rabbit	50081	CST
OGG1/2	1:2500(WB)	Mouse	sc-376935	Santa Cruz
β-actin	1:2500(WB)	Rabbit	AC026	Abclonal
FNDC5	1:2500(WB)	Rabbit	ab174833	Abcam
TUJ1	1:2500(WB)	Rabbit	G712A	Promega
GFAP	1:2500(WB)	Rabbit	A0237	Abclonal
OLIGO2	1:2500(WB)	Rabbit	A12814	Abclonal
IBA1	1:2500(WB)	Rabbit	A1527	Abclonal
6X His tag	1:2500(WB)	Rabbit	ab18184	Abcam
Alexa Fluor 488 Anti-6X His tag	1:1000(IF)	Rabbit	ab237336	Abcam

Supplementary Information: full, uncropped main western blot

Figure 1 e

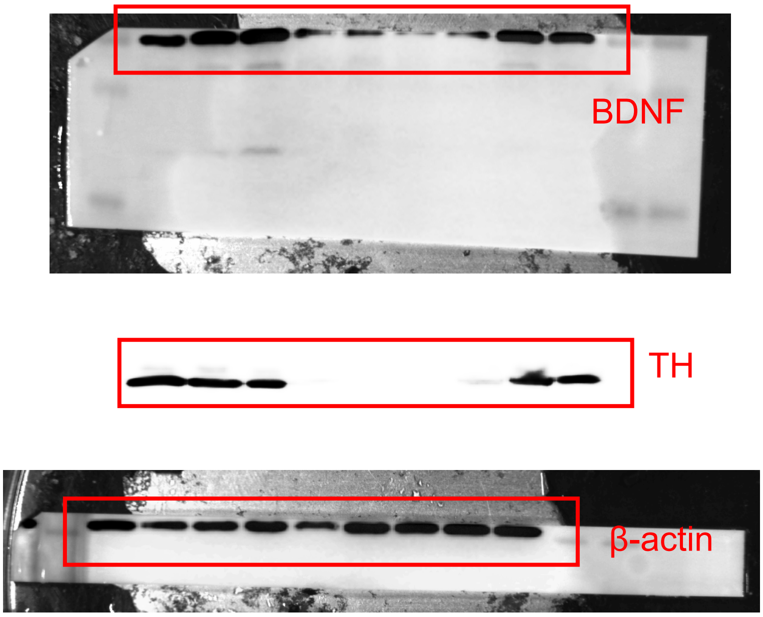

Figure 1 q

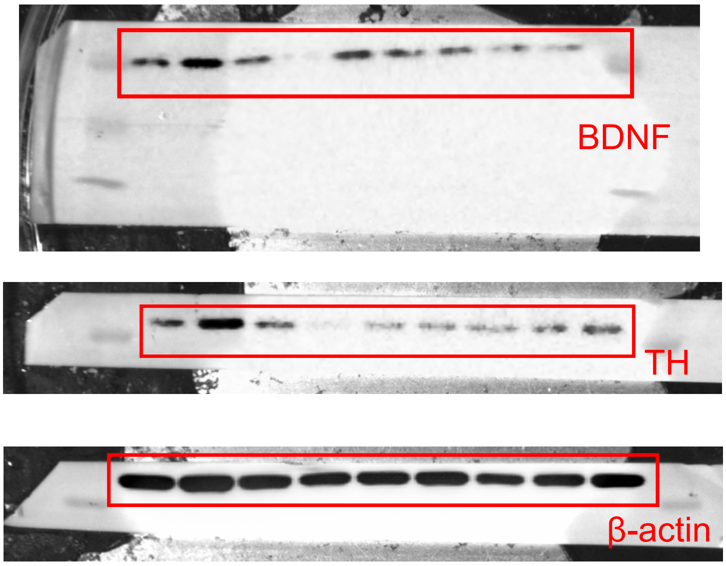

Figure 1 k

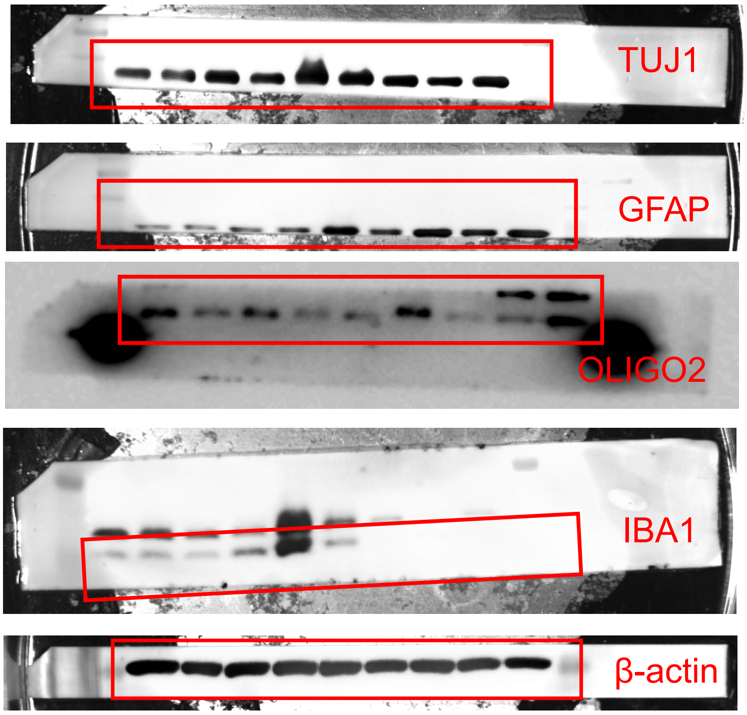

Figure 1 w

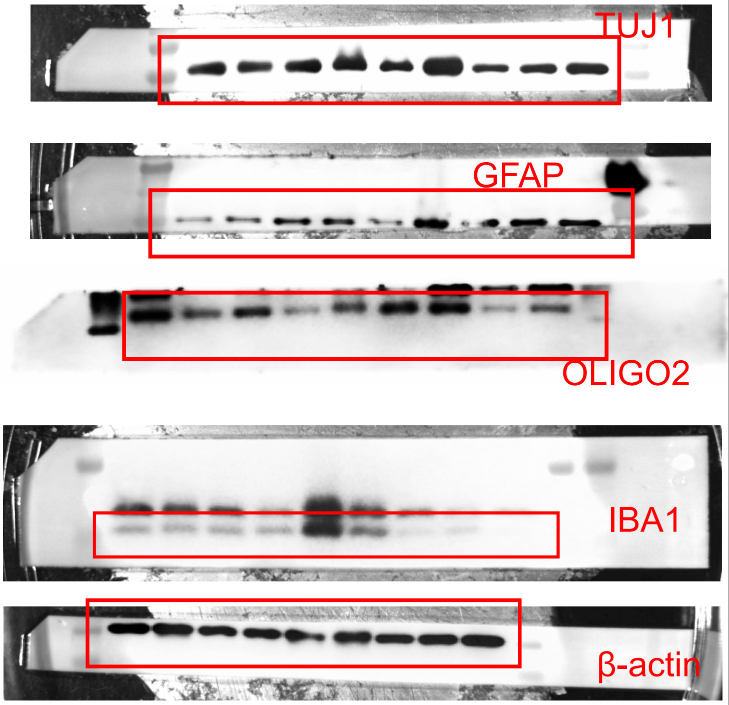

Figure 2 a

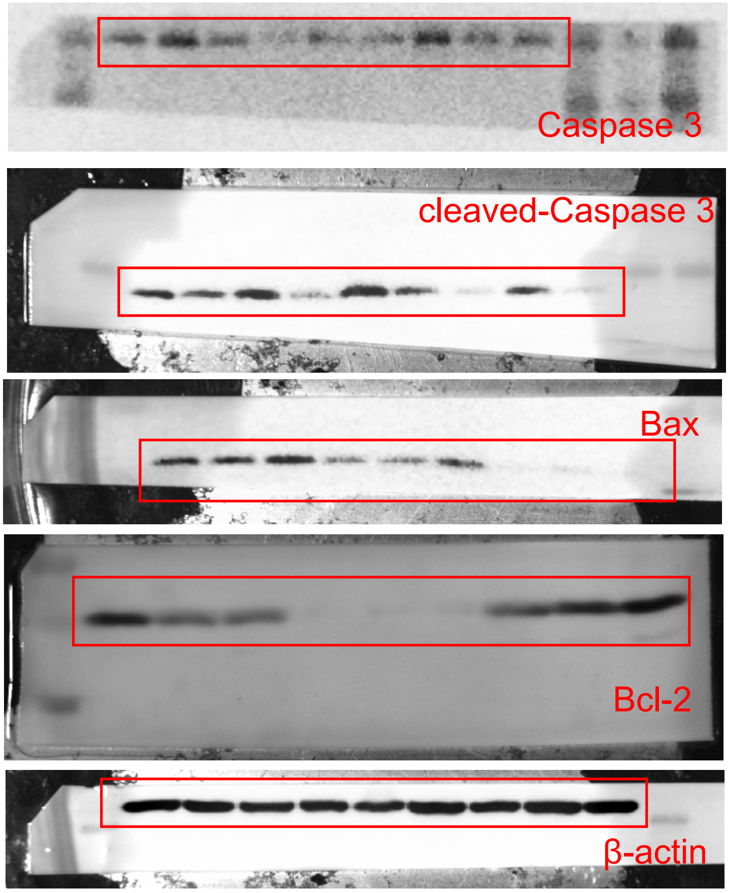

Figure 2 c

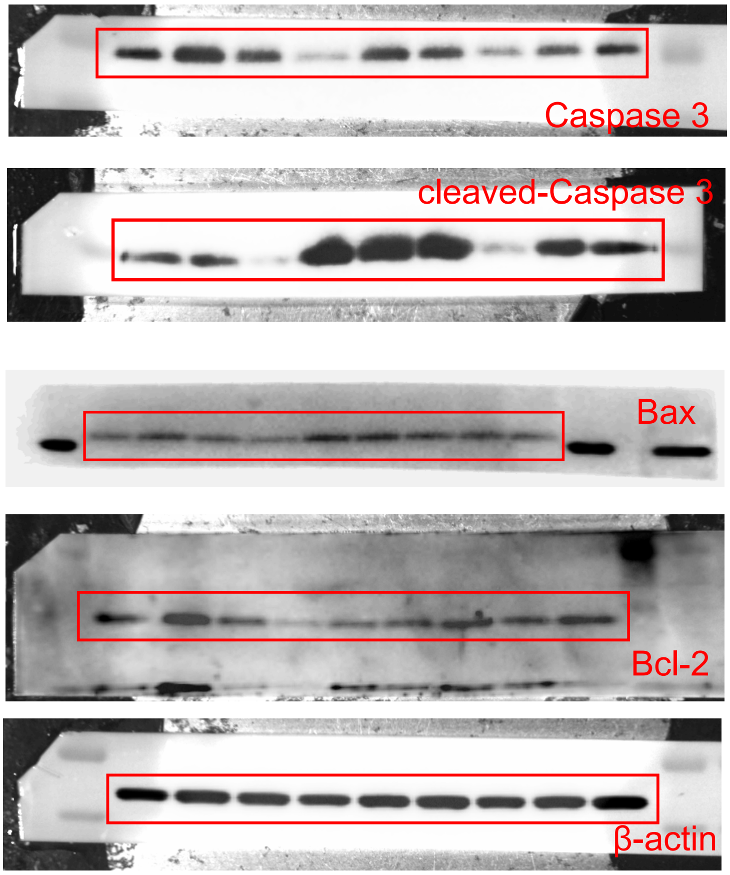

Figure 2 i

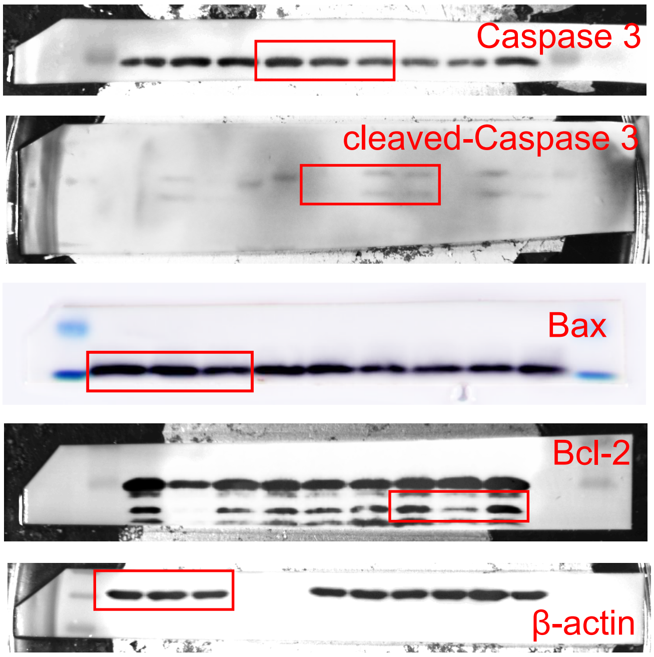

Figure 2 k

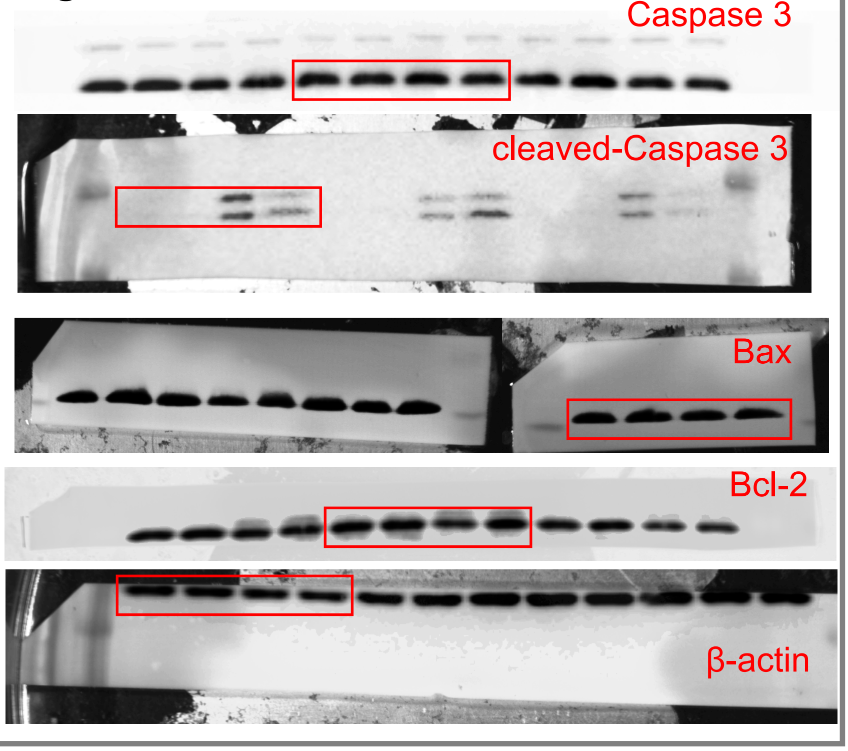

Figure 3 g

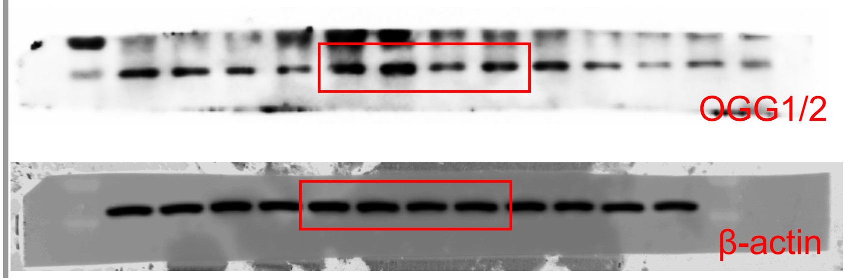

Figure 3 i

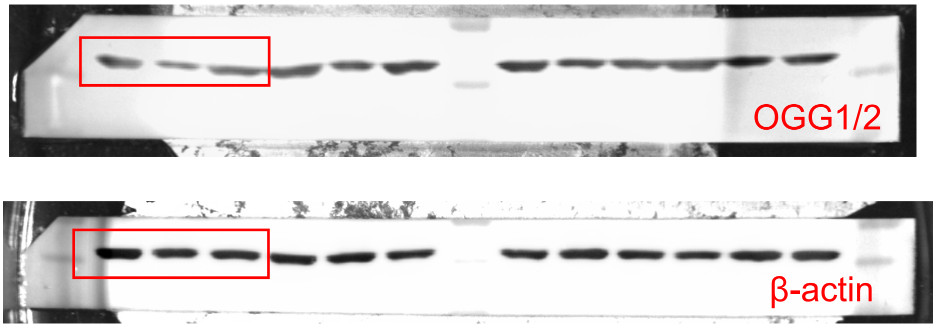

Figure 4 g

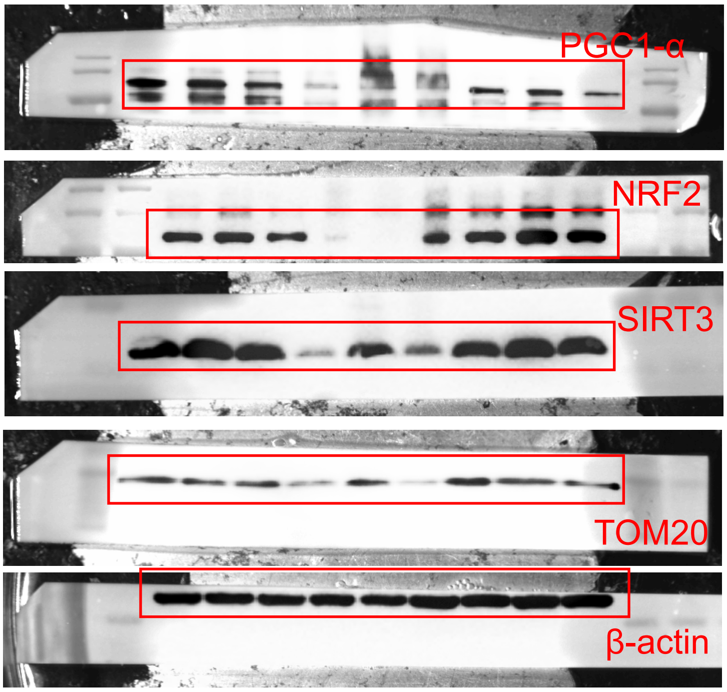

Figure 4 i

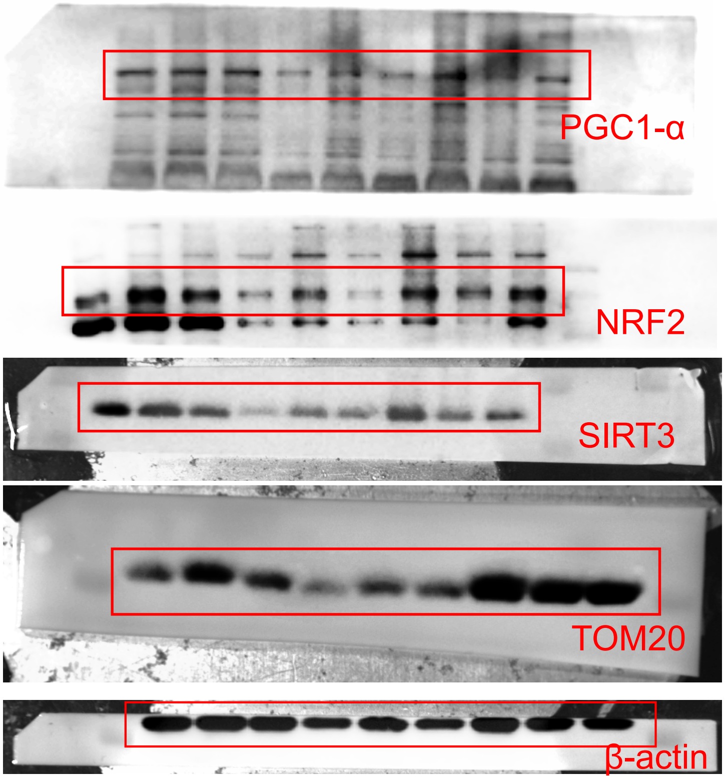

Figure 4 n

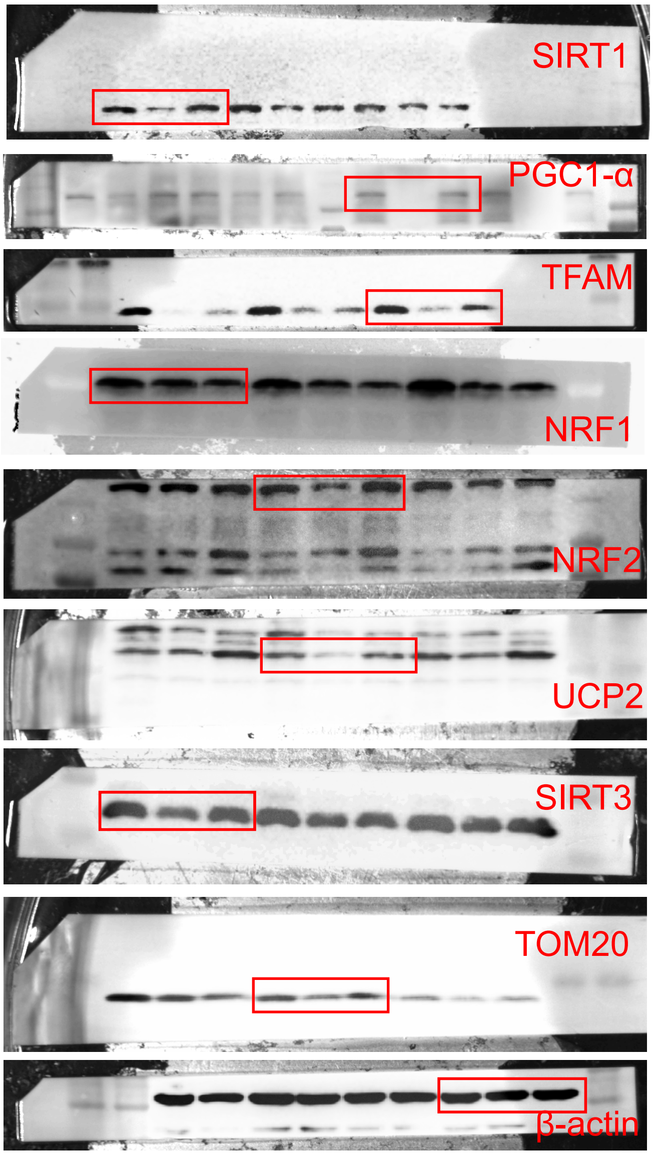

Figure 4 p

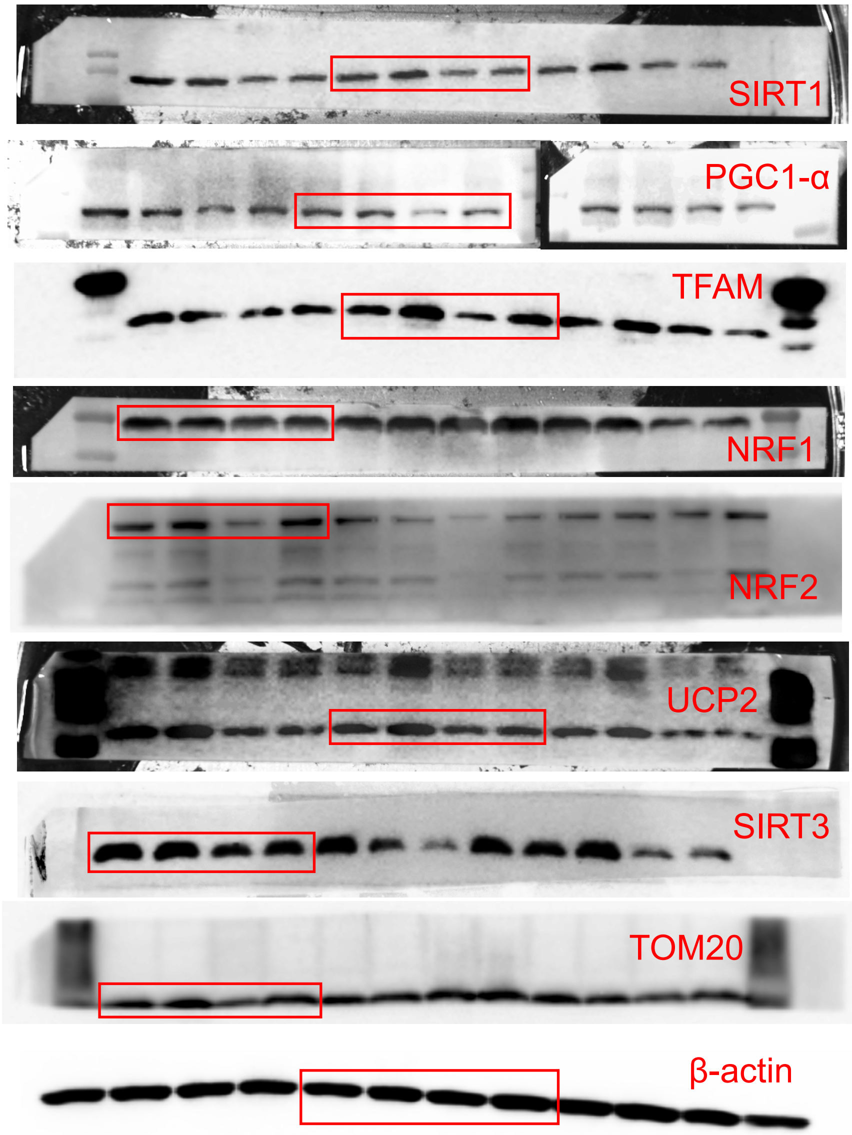

Figure 5 a

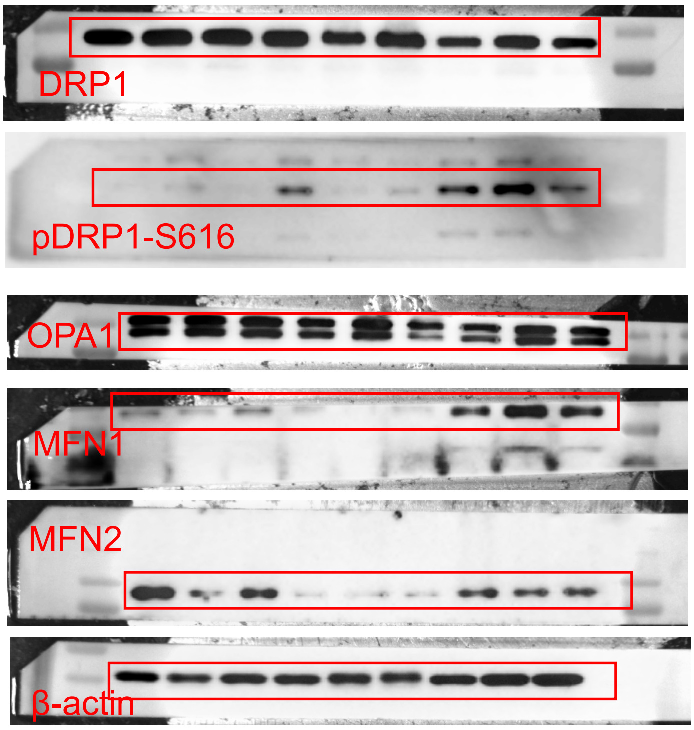

Figure 5 c

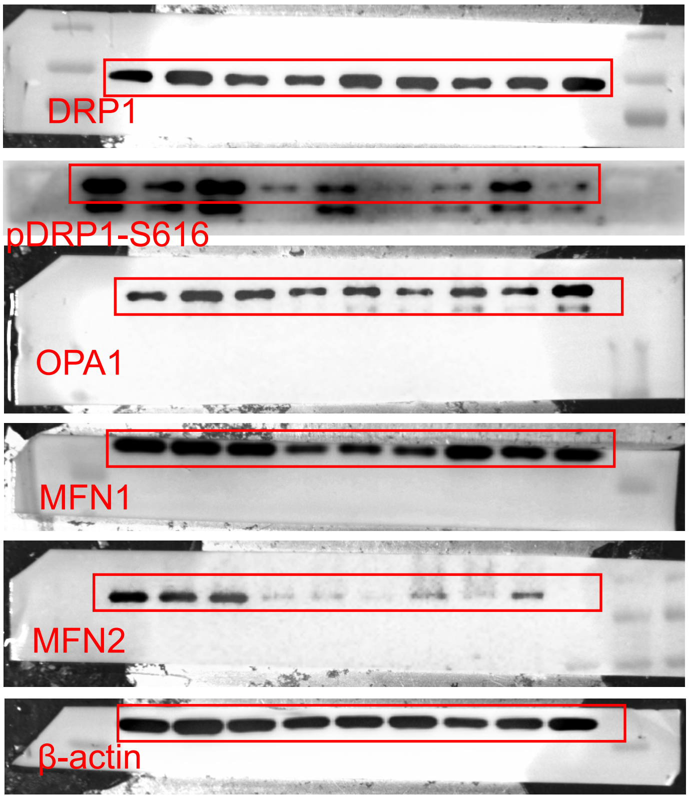

Figure 5 k

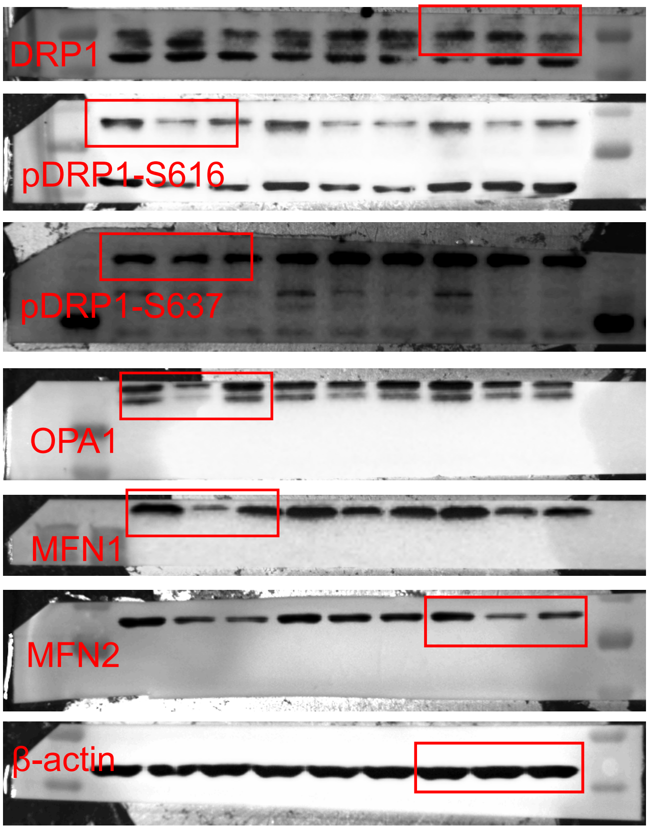

Figure 5 m

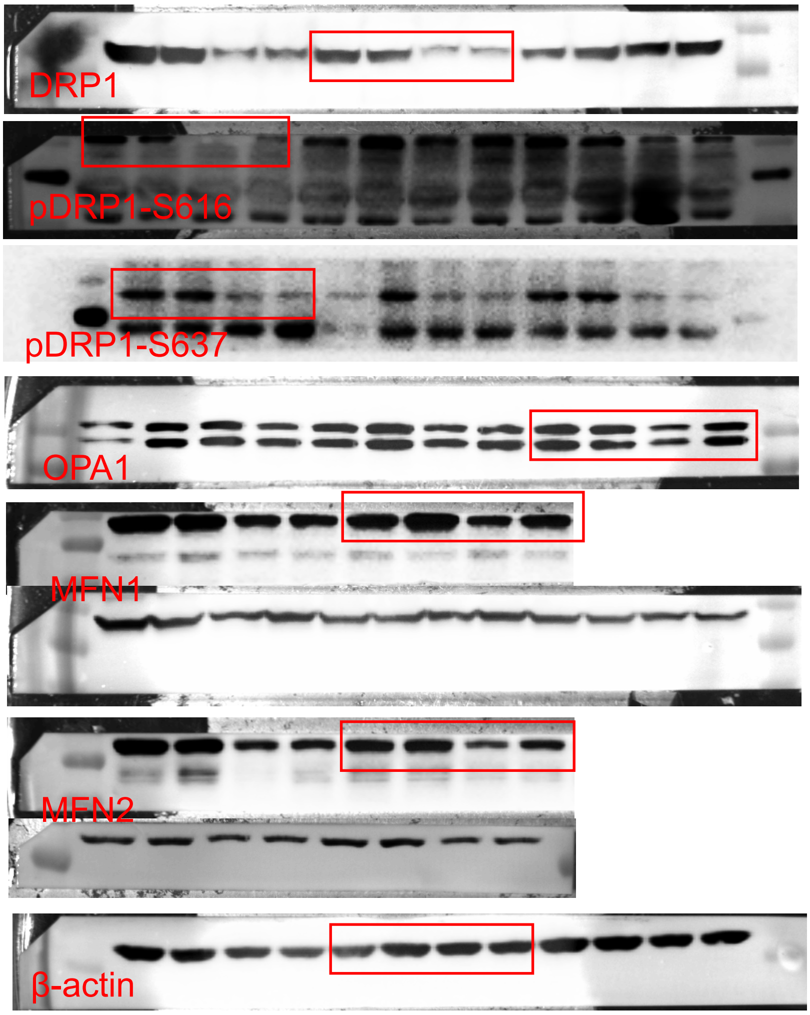

Figure 6 a

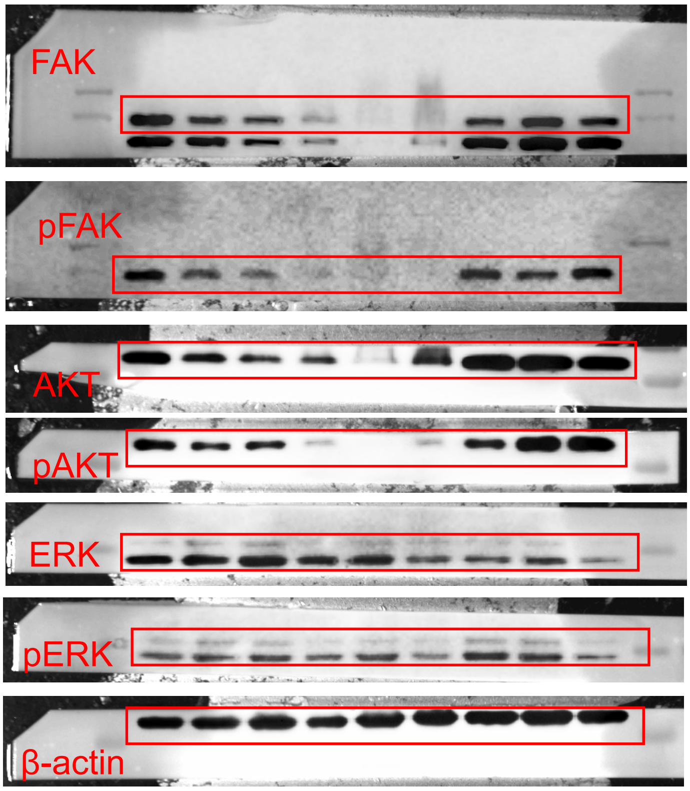

Figure 6 c

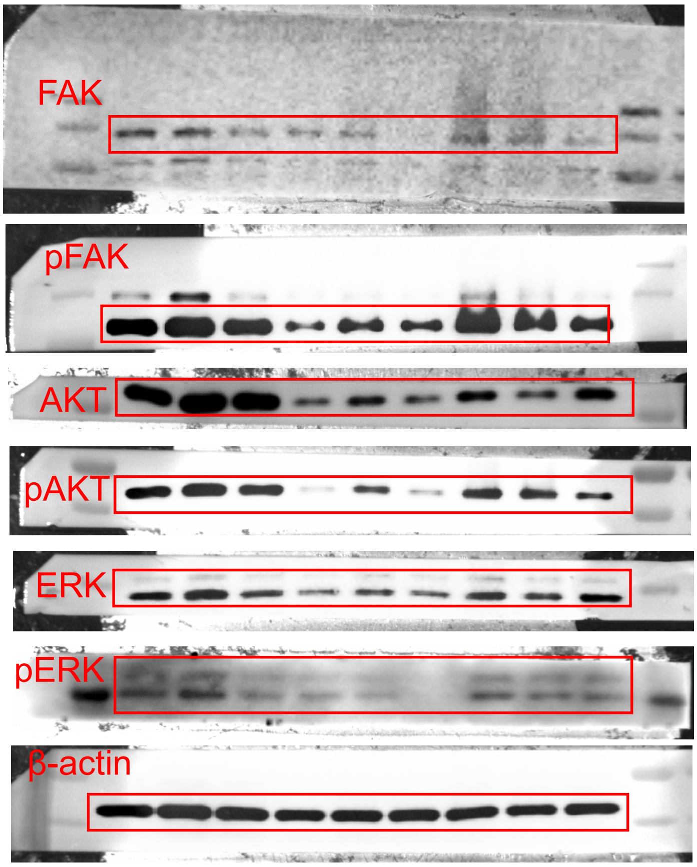

Figure 6 e

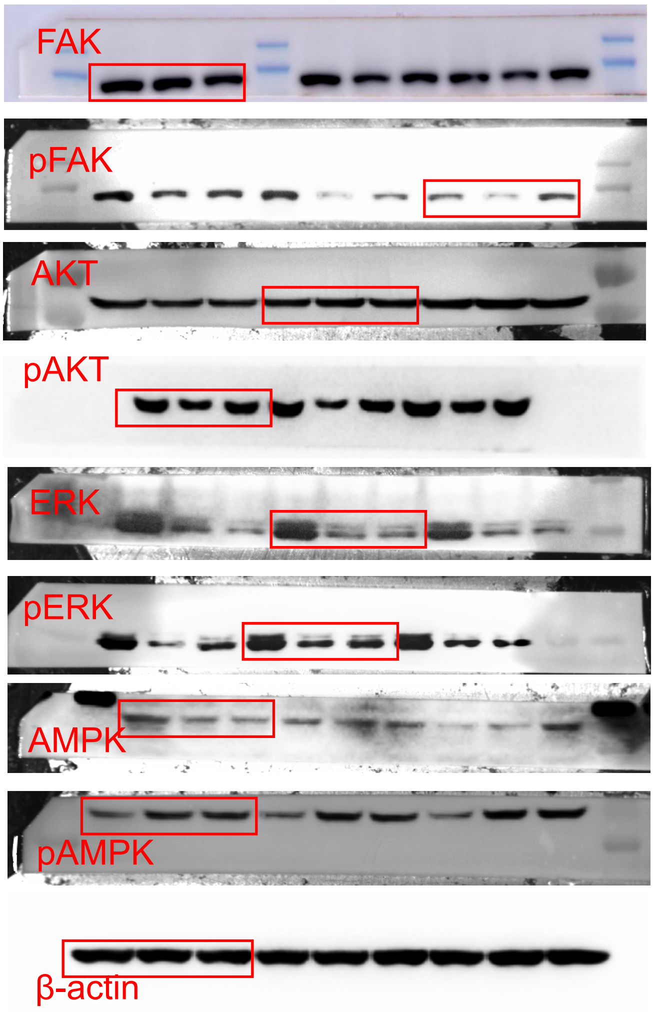

Figure 6 g

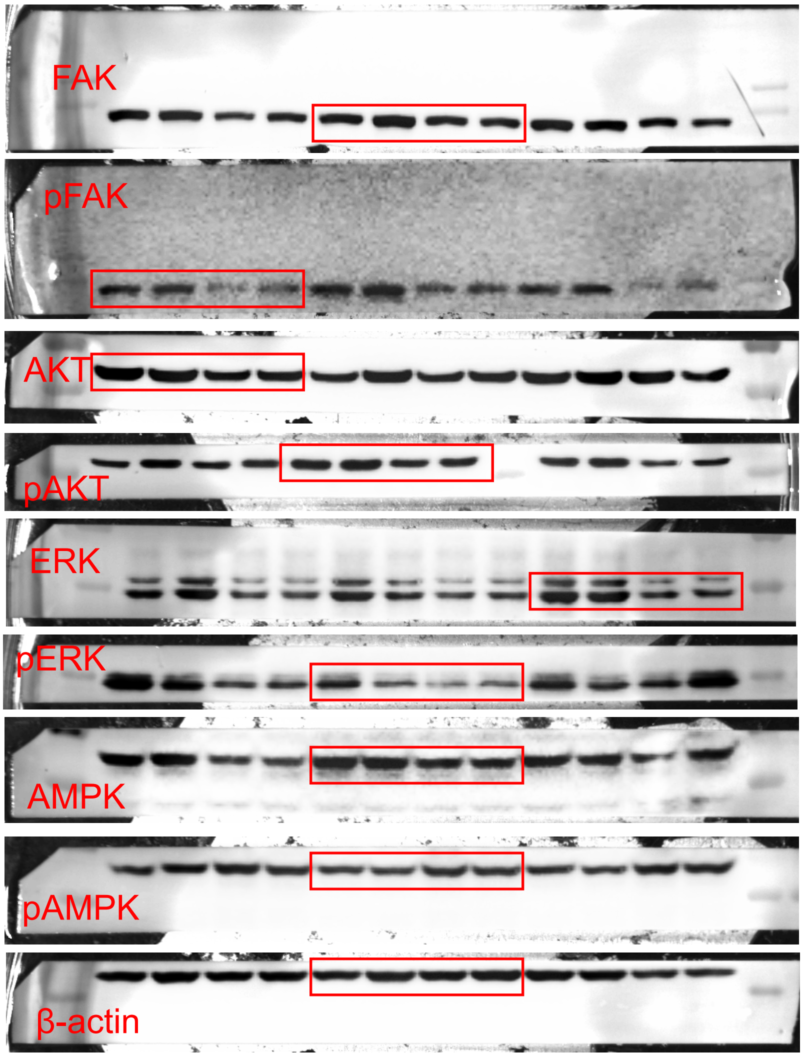

Figure 7 f

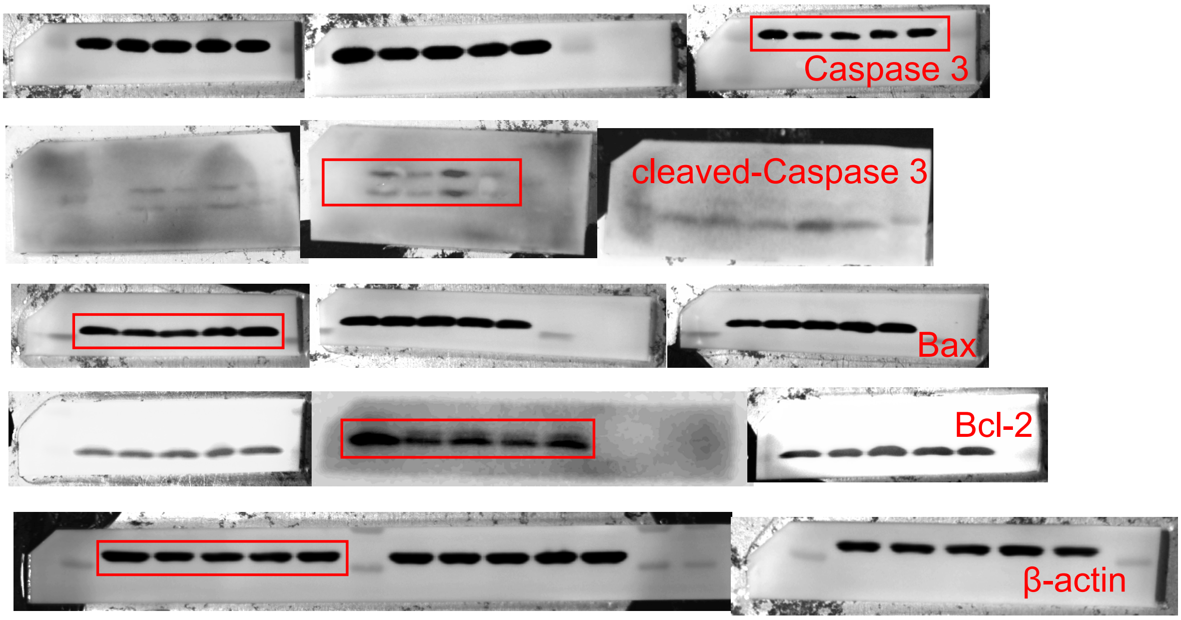

Figure 7 h

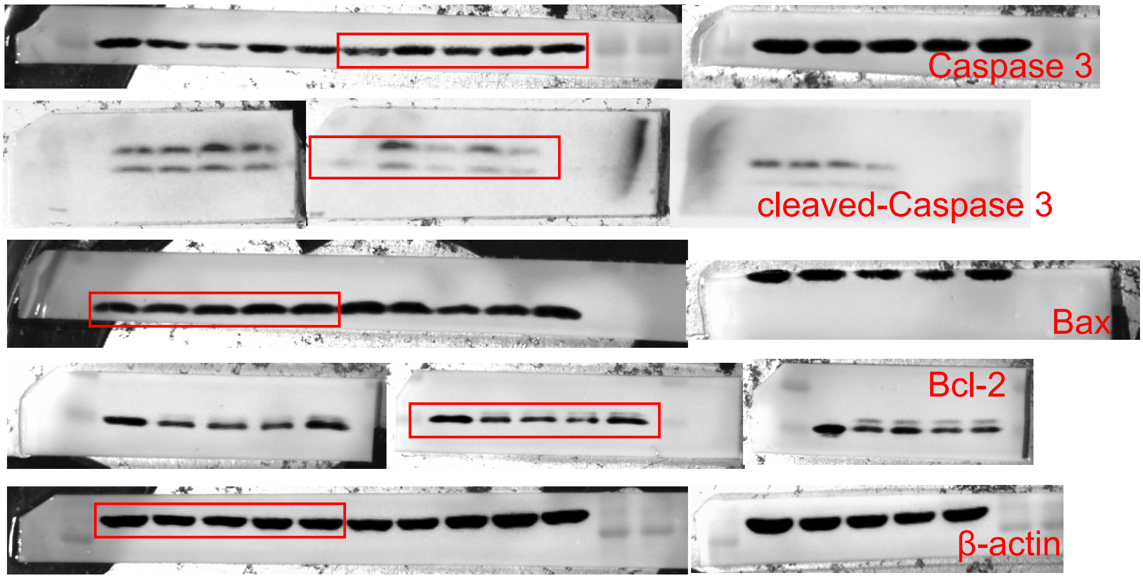

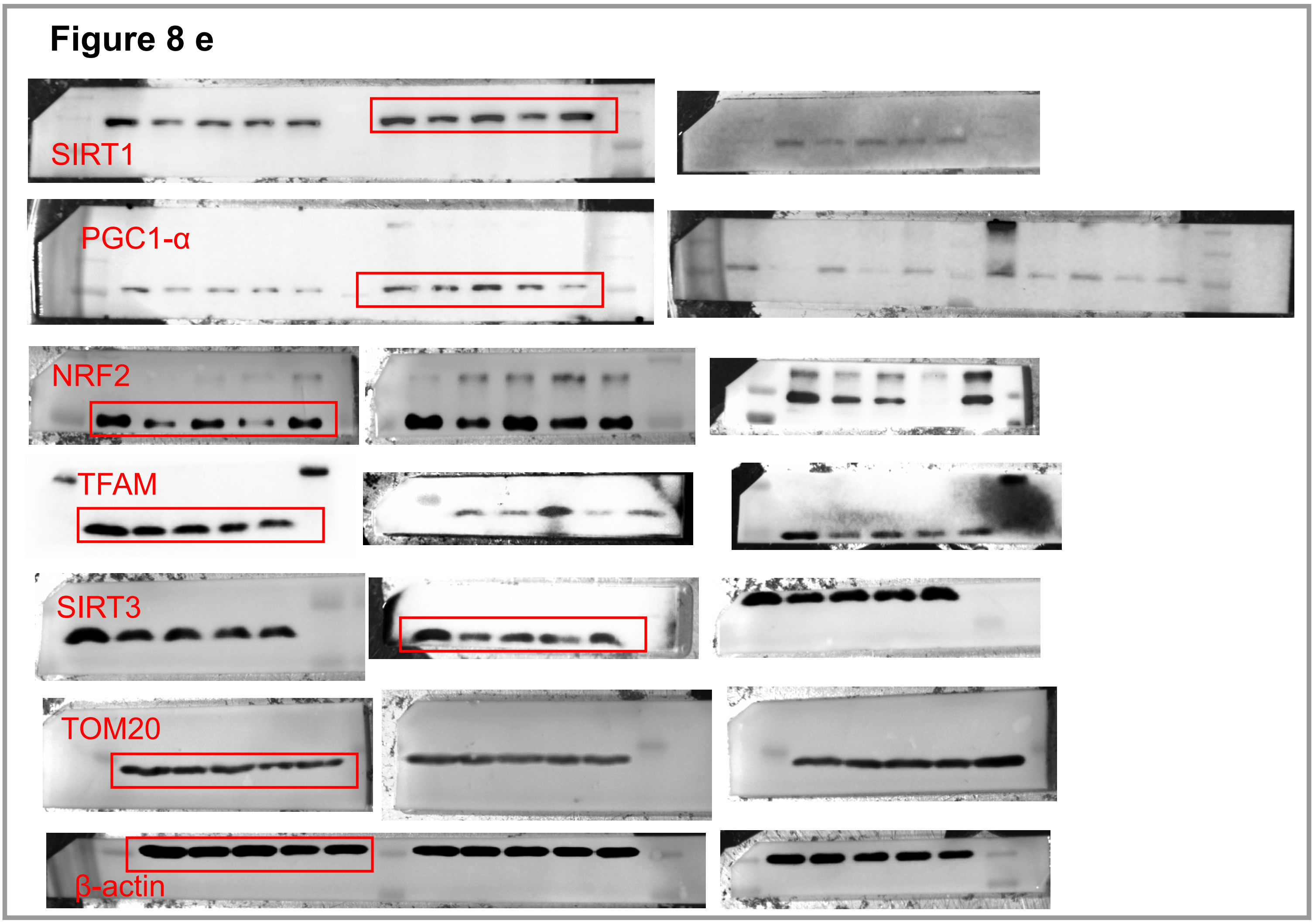
Figure 8 e
SIRT1
PGC1-α
NRF2
TFAM
SIRT3
TOM20
β-actin

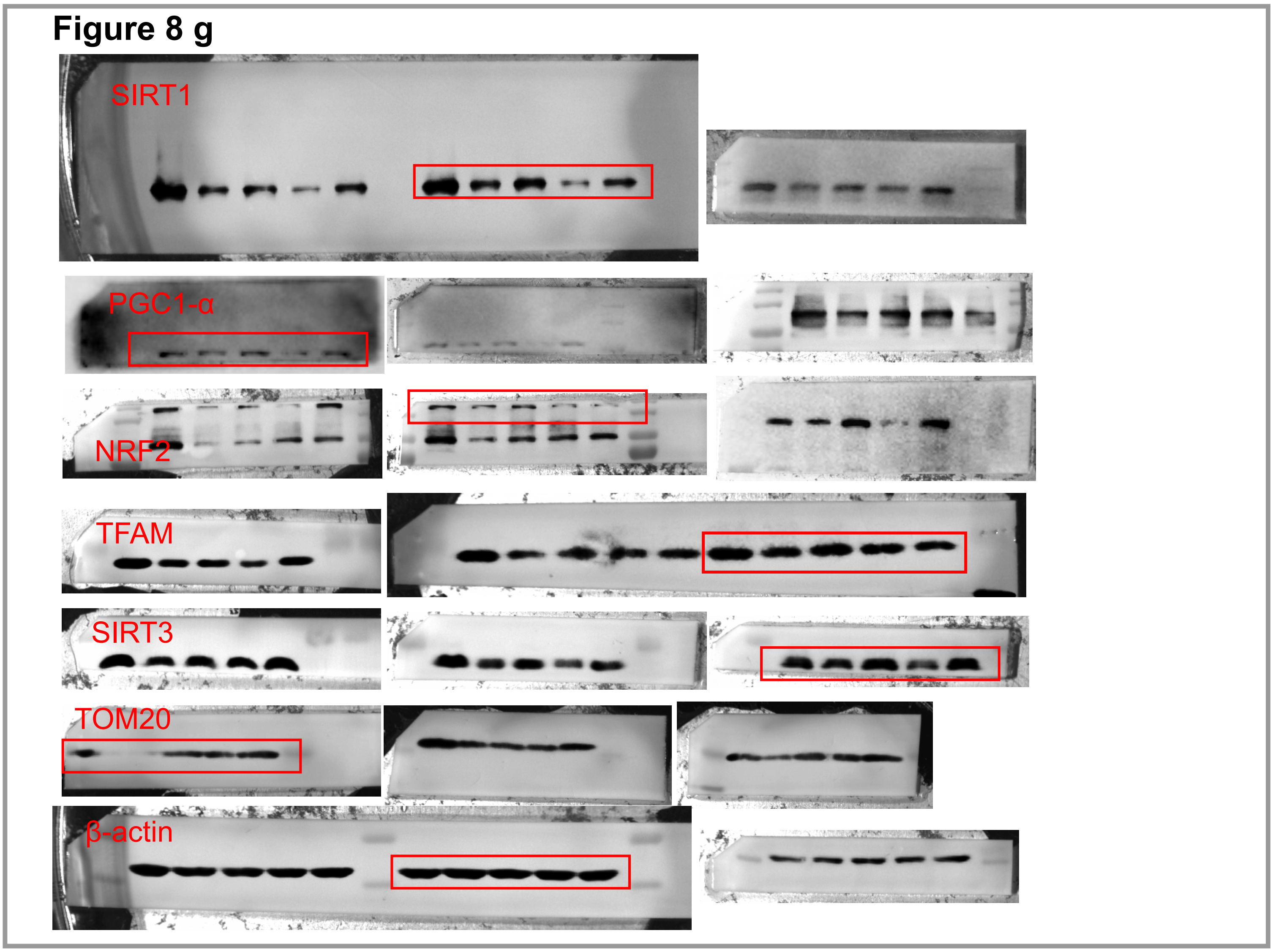
Figure 8 g
SIRT1
PGC1-α
NRF2
TFAM
SIRT3
TOM20
β-actin

Figure 9 e

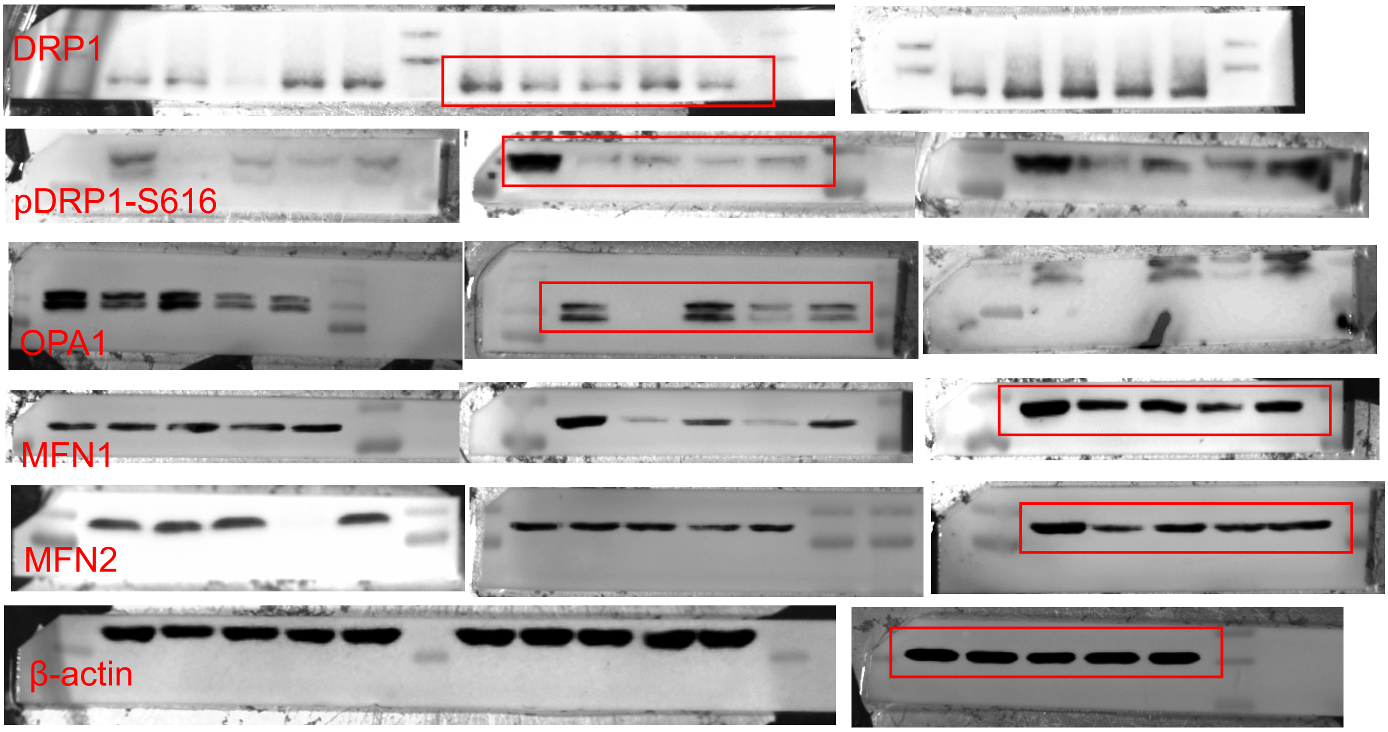

Figure 9 g

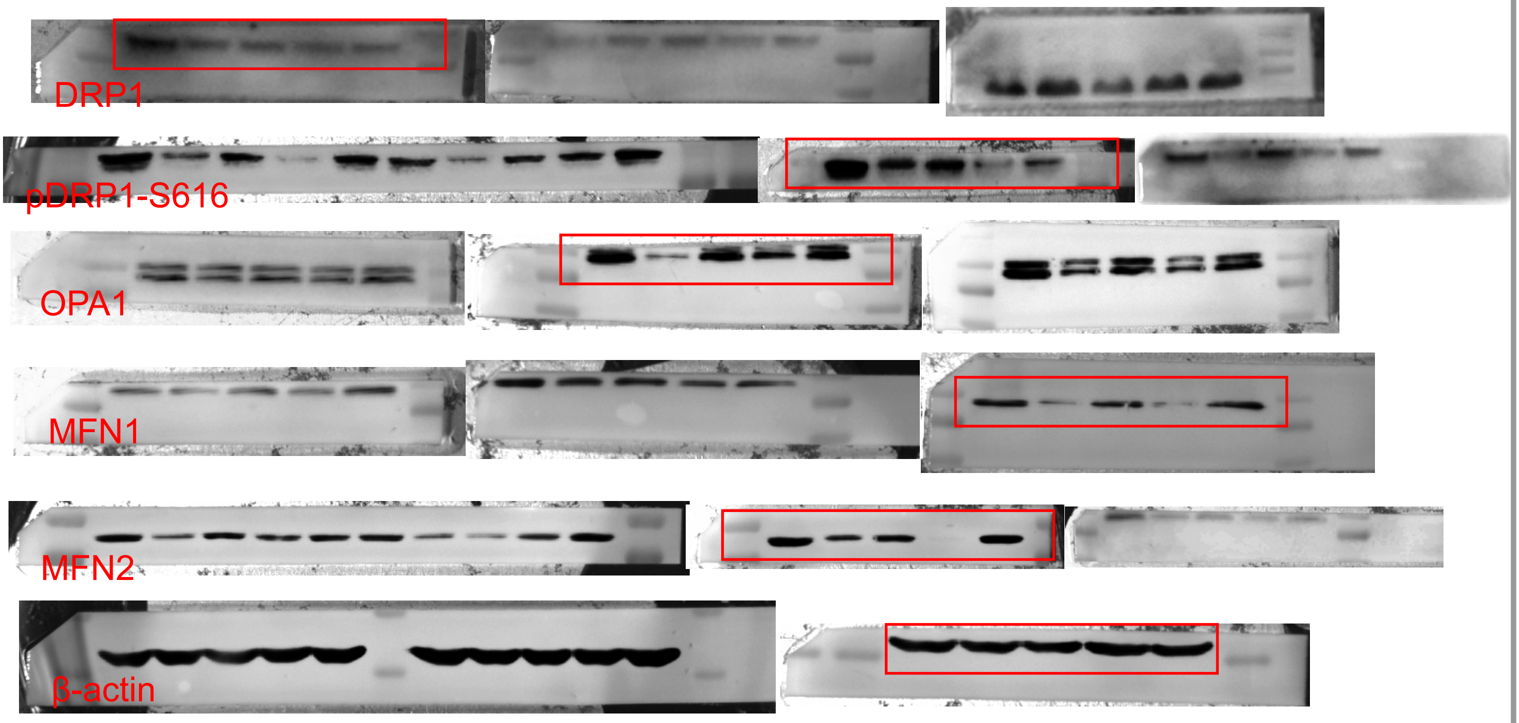

Figure 10 c

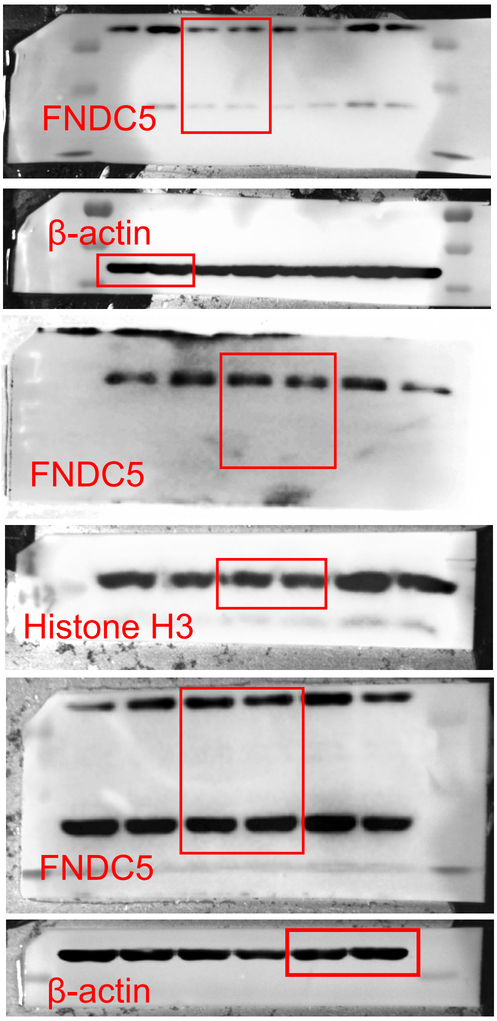

Figure 10 d

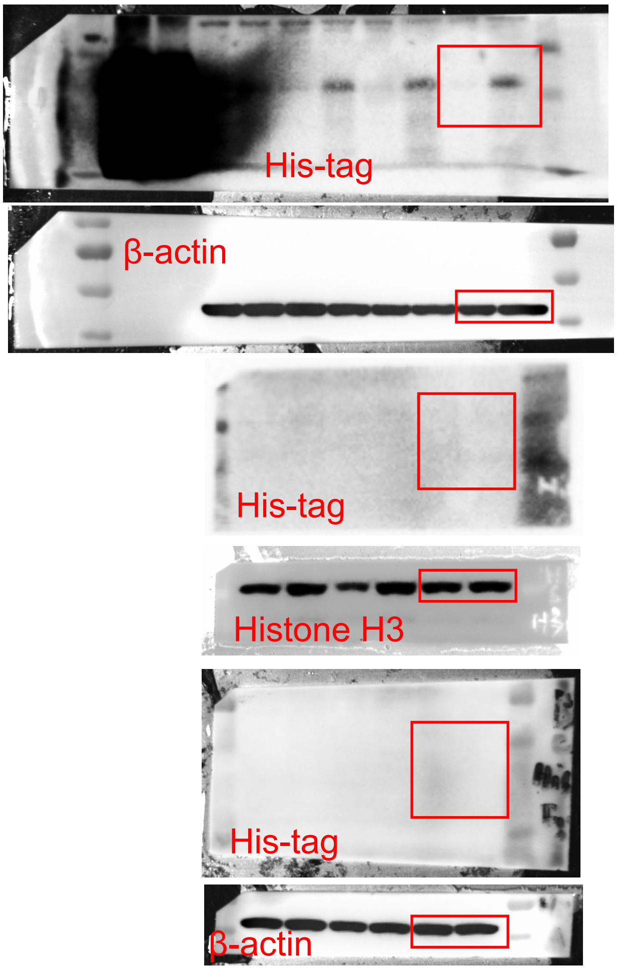

Figure 10 g

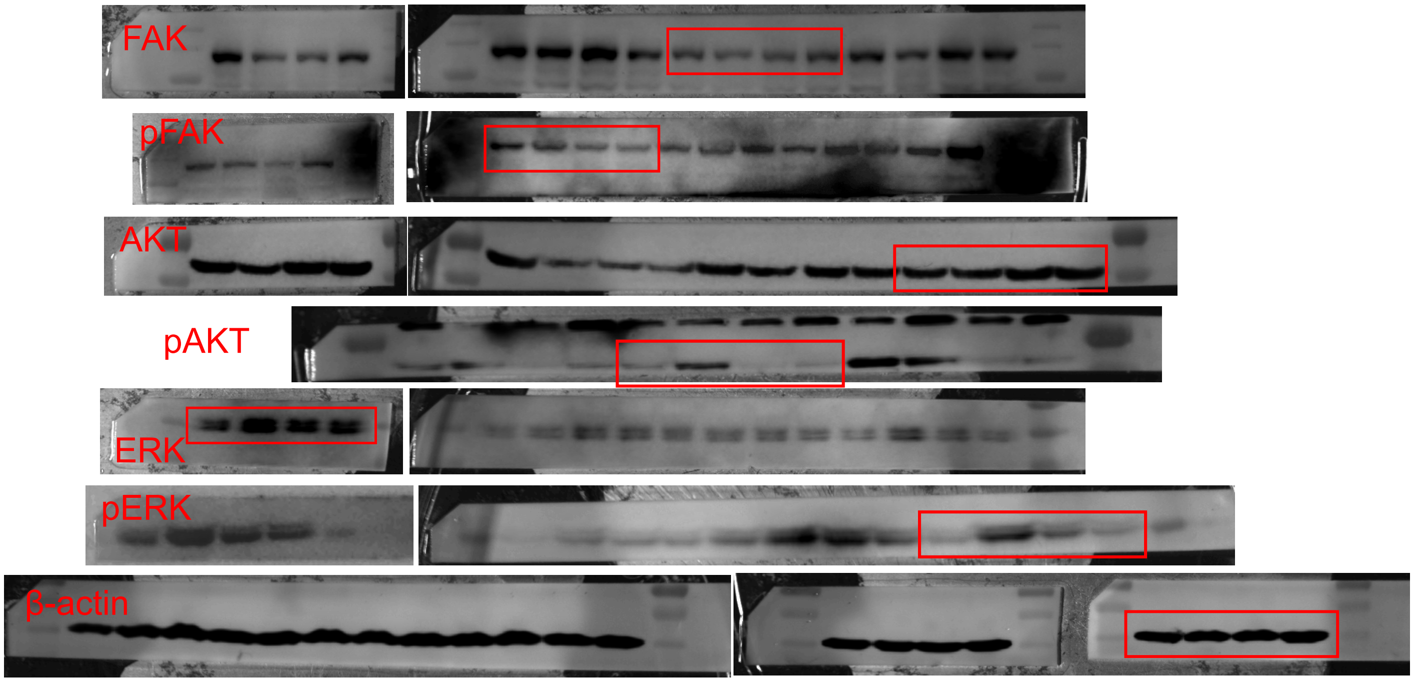

Supplementary Figure 4 e

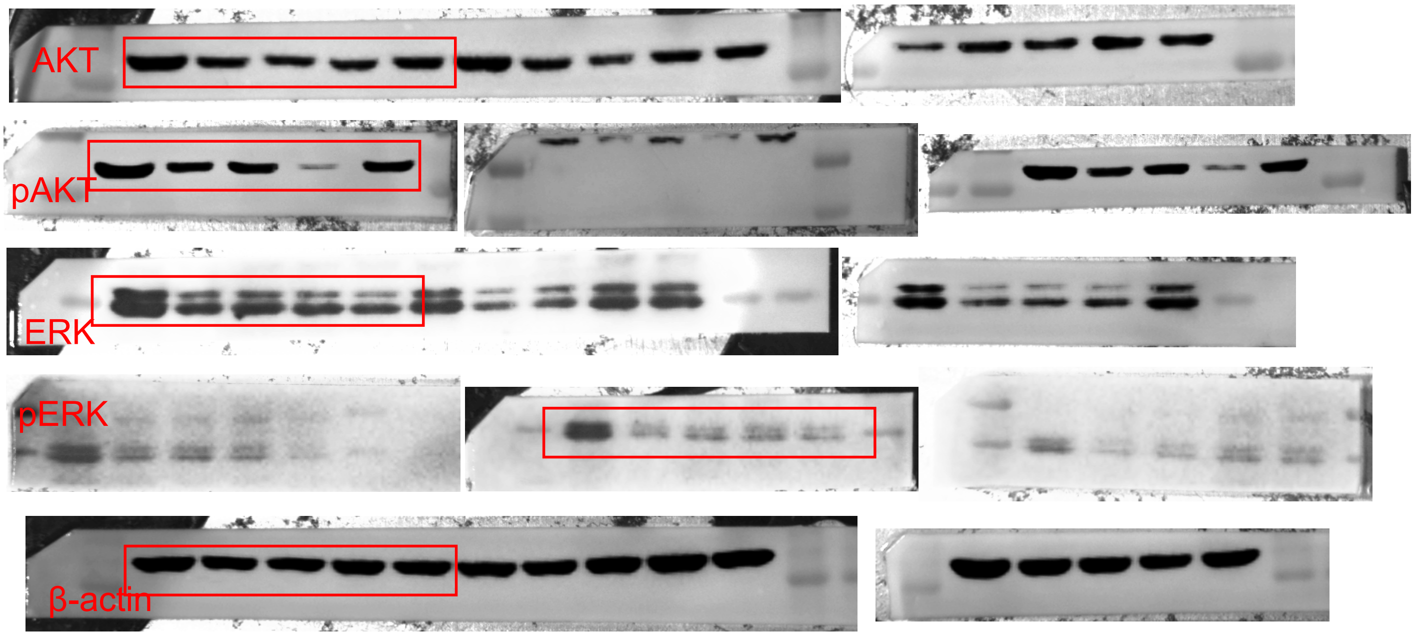

Supplementary Figure 4 g

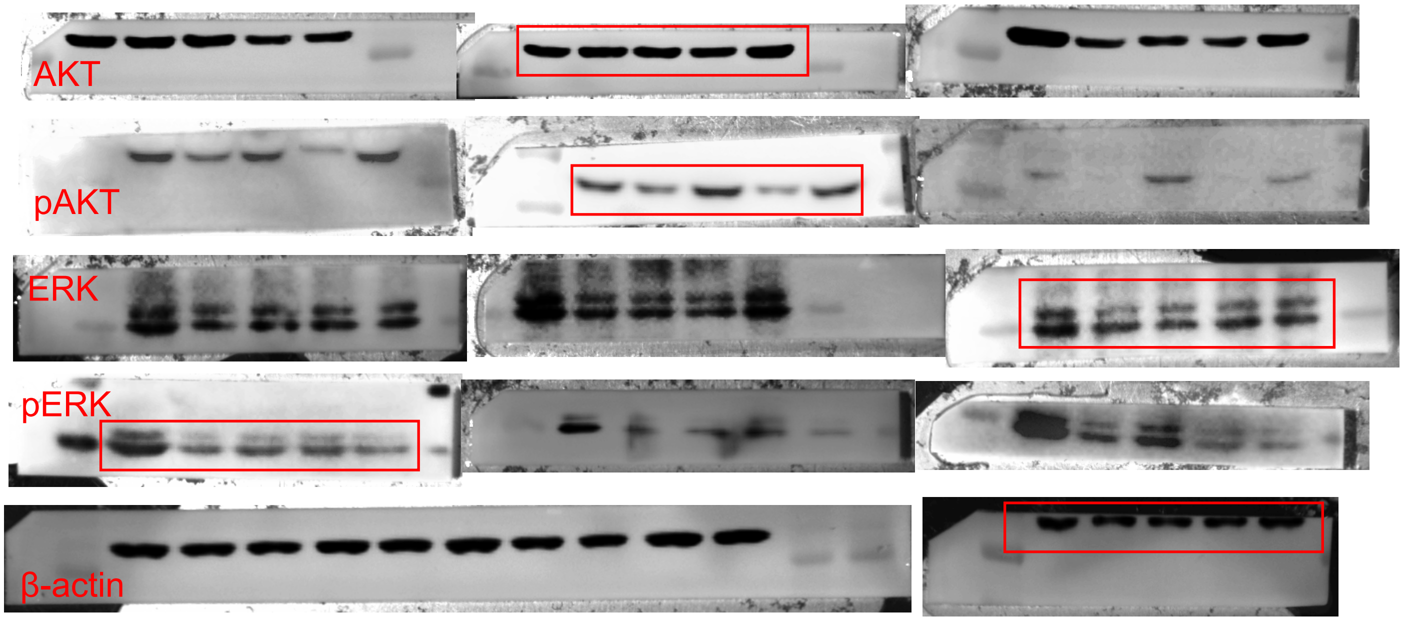

Supplement: Supplementary file 1 — Supplementary materials [file 41531_2023_453_MOESM1_ESM.pdf]
