# Supplementary material for: Irisin exhibits neuroprotection by preventing mitochondrial damage in Parkinson’s disease
Source: NPJ Parkinsons Dis. 2023 Jan 31;9:13. doi: 10.1038/s41531-023-00453-9 (PMC9889817; doi:10.1038/s41531-023-00453-9)
Supplement: Supplementary file 2 — Reporting Summary [file 41531_2023_453_MOESM2_ESM.pdf]

nature portfolio

Corresponding author(s): Lingjing Jin; Siguang Li

Last updated by author(s): Dec 30, 2022

Reporting Summary

Nature Portfolio wishes to improve the reproducibility of the work that we publish. This form provides structure for consistency and transparency in reporting. For further information on Nature Portfolio policies, see our Editorial Policies and the Editorial Policy Checklist.

Statistics

For all statistical analyses, confirm that the following items are present in the figure legend, table legend, main text, or Methods section.

n/a	Confirmed	
☐	☒	The exact sample size (*n*) for each experimental group/condition, given as a discrete number and unit of measurement
☐	☒	A statement on whether measurements were taken from distinct samples or whether the same sample was measured repeatedly
☐	☒	The statistical test(s) used AND whether they are one- or two-sided<br>*Only common tests should be described solely by name; describe more complex techniques in the Methods section.*
☒	☐	A description of all covariates tested
☐	☒	A description of any assumptions or corrections, such as tests of normality and adjustment for multiple comparisons
☐	☒	A full description of the statistical parameters including central tendency (e.g. means) or other basic estimates (e.g. regression coefficient) AND variation (e.g. standard deviation) or associated estimates of uncertainty (e.g. confidence intervals)
☐	☒	For null hypothesis testing, the test statistic (e.g. *F*, *t*, *r*) with confidence intervals, effect sizes, degrees of freedom and *P* value noted<br>*Give P values as exact values whenever suitable.*
☒	☐	For Bayesian analysis, information on the choice of priors and Markov chain Monte Carlo settings
☒	☐	For hierarchical and complex designs, identification of the appropriate level for tests and full reporting of outcomes
☐	☒	Estimates of effect sizes (e.g. Cohen's *d*, Pearson's *r*), indicating how they were calculated

Our web collection on statistics for biologists contains articles on many of the points above.

Software and code

Policy information about availability of computer code

Data collection	For bulk RNA sequencing, the fresh brain tissue of mice was collected after perfusing. Nigral tissues were manually extracted and instantly froze in liquid nitrogen. All samples were subjected to RNA-seq by the LC Bio (Zhejiang, China) and data analysis.
Data analysis	Illumina NovaSeq 6000; R studio; R i386 4.1.2; edge package

For manuscripts utilizing custom algorithms or software that are central to the research but not yet described in published literature, software must be made available to editors and reviewers. We strongly encourage code deposition in a community repository (e.g. GitHub). See the Nature Portfolio guidelines for submitting code & software for further information.

Data

Policy information about availability of data

All manuscripts must include a data availability statement. This statement should provide the following information, where applicable:

- Accession codes, unique identifiers, or web links for publicly available datasets
- A description of any restrictions on data availability
- For clinical datasets or third party data, please ensure that the statement adheres to our policy

RNA-Seq datasets generated here are uploaded to NCBI Gene Expression Omnibus (GEO) database (GSE number: GSE214542, https://www.ncbi.nlm.nih.gov/geo/query/acc.cgi?acc=GSE214542). Extra data are available from the corresponding author upon request.

Human research participants

Policy information about studies involving human research participants and Sex and Gender in Research.

Reporting on sex and gender	The subjects included 15 males and 8 females. The study did not divide people into groups based on gender.
Population characteristics	1. The mean age of recruited subjects was 68.08 years (±7.07 years) and they were diagnosed with Parkinson's disease according to the clinical diagnostic criteria of Parkinson's Disease UK Brain Bank.<br>2. Genotype information of recruited subjects is unknown.<br>3. Recruited subjects received oral levodopa treatment (310.79±206.89mg/day).
Recruitment	In this study, we included 23 patients with PD, who accepted exercise classes from June 2014 to December 2015 in Shanghai Tongji Hospital, Tongji University School of Medicine. The inclusion criteria were as follows: (i) recruited patients of PD were evaluated by neurologists using the United Kingdom Parkinson's Disease Society Brain Bank clinical diagnostic criteria; (ii) patients were asked to follow the original medication dosage during the entire study; (iii) patients accomplished exercise twice a week for 12 weeks as described in the previous study; (iv) the effect of exercise on PD was assessed by Parkinson's Disease Rating Scale Motor Examination (UPDRSIII), and the Berg Balance Scale (BBS); (v) assessment was completed by clinicians who were blinded to the mode of exercise intervention before and at the end of the 12th weeks ; (vi) blood samples of patients in fasting state were collected by a standard procedure for RNA isolation before the start and 48–72h after the completion of the 12-week training program, and serum samples that was isolated immediately were preserved for long-term storage at −80◦C. The exclusion criteria were that patients had other medical conditions.
Ethics oversight	The Ethics Committee of Shanghai Tongji Hospital

Note that full information on the approval of the study protocol must also be provided in the manuscript.

Field-specific reporting

Please select the one below that is the best fit for your research. If you are not sure, read the appropriate sections before making your selection.

☒ Life sciences ☐ Behavioural & social sciences ☐ Ecological, evolutionary & environmental sciences

For a reference copy of the document with all sections, see nature.com/documents/nr-reporting-summary-flat.pdf

Life sciences study design

All studies must disclose on these points even when the disclosure is negative.

Sample size	In clinical researches, sample size was estimated by PASS software.<br>The experimental sample size was estimated by the degree of freedom (E) of variance analysis. E= sum of experimental animals in each group - number of groups (the value of E should range from 10 to 20).
Data exclusions	In clinical researches, the exclusion criteria were that patients had other medical conditions.<br>In animal and cell researches, all data are presented as mean ± standard deviation (SD). If one measured value was greater than mean + triple SD and was less than mean - triple SD, the measured value was regarded as outlier and was eliminated.
Replication	In animals and cells researches, each experiment was replicated at least three times.
Randomization	1. In this study, we used the self-contrasted method. Thus, participants were not allocated into experimental groups.<br>2. Organisms were ranked and numbered based on behavioral assessments, then they were grouped using a random-number generation program and accepted corresponding drug treatments.
Blinding	During data collection, the assessment of UPDRSIII and BBS was completed by clinicians who were blinded to the mode of exercise intervention before and at the end of the 12th weeks.<br>During data analysis, data groups were referred to as random numbers, and the grouping status is unknown to analysts.

Reporting for specific materials, systems and methods

We require information from authors about some types of materials, experimental systems and methods used in many studies. Here, indicate whether each material, system or method listed is relevant to your study. If you are not sure if a list item applies to your research, read the appropriate section before selecting a response.

Materials & experimental systems

n/a	Involved in the study	
☐	☒	Antibodies
☒	☐	Eukaryotic cell lines
☒	☐	Palaeontology and archaeology
☐	☒	Animals and other organisms
☐	☒	Clinical data
☒	☐	Dual use research of concern

Methods

n/a	Involved in the study	
☒	☐	ChIP-seq
☒	☐	Flow cytometry
☒	☐	MRI-based neuroimaging

Antibodies

Antibodies used	All antibodies used in the study were listed in supplementary table 1.
Validation	All antibodies used in the study were listed in supplementary table 1.

Animals and other research organisms

Policy information about studies involving animals; ARRIVE guidelines recommended for reporting animal research, and Sex and Gender in Research

Laboratory animals	Male C57BL/6 mice (6-8 weeks old, weighing 20–30 g) were purchased from Shanghai SLAC Laboratory Animal Company.
Wild animals	The study did not involve wild animals.
Reporting on sex	Findings apply to only one sex (male).
Field-collected samples	Mice were housed (5 mice per cage) in the Tongji University Animal House Facility (Shanghai, China), in light (12 h light/dark) and temperature (20–22 °C).
Ethics oversight	The National Institutes of Health Guide for the Care and Use of Laboratory Animals<br>The ethics committee for the use of experimental animals in Tongji University

Note that full information on the approval of the study protocol must also be provided in the manuscript.

Clinical data

Policy information about clinical studies

All manuscripts should comply with the ICMJE guidelines for publication of clinical research and a completed CONSORT checklist must be included with all submissions.

Clinical trial registration	ChiCTR-TRC-14004707
Study protocol	http://www.chictr.org.cn/showproj.aspx?proj=4866
Data collection	In this study, we included 23 patients with PD, who accepted exercise classes from June 2014 to December 2015 in Shanghai Tongji Hospital, Tongji University School of Medicine.
Outcomes	We used Parkinson's Disease Rating Scale Motor Examination (UPDRSIII), and the Berg Balance Scale (BBS) as pre-defined primary and secondary out come. Assessment was completed by clinicians who were blinded to the mode of exercise intervention before and at the end of the 12th weeks.
